# Evaluation of a Faith‐Placed Health Education Service on Bowel Cancer Screening in Mosques in East London

**DOI:** 10.1111/hex.70006

**Published:** 2024-08-24

**Authors:** Salman Waqar, Dharani Yerrakalva, Thomas E. Duffy, Jake Chambers, Zohra Ali, Paul Thomas, Caroline Cook, Sufia Alam, Leena Khagram, Samantha Quaife, Stephen W. Duffy

**Affiliations:** ^1^ Department of Primary Care and Public Health Imperial College London London UK; ^2^ Wolfson Institute of Population Health Queen Mary University of London London UK; ^3^ North East London Cancer Alliance London UK; ^4^ Royal Marsden NHS Foundation Trust London UK; ^5^ East London Mosque London UK

**Keywords:** bowel cancer, inequalities, Muslim, screening, uptake

## Abstract

**Background:**

Bowel cancer screening using faecal immunochemical testing is provided in the United Kingdom with the aim of reducing mortality from colorectal cancer. However, there are low participant rates among ethnic minorities across the United Kingdom. Faith‐placed interventions have the potential to improve screening rates among such populations, but studies examining their effectiveness are scarce.

**Methods:**

We delivered a presentation on bowel cancer screening to 204 Muslims in seven mosques in East London (intervention group). All participants completed a questionnaire regarding attitudes, perceptions and knowledge of bowel cancer screening before and after the presentation. Concurrently, we administered the questionnaire to 72 subjects attending a mosque that did not receive the presentation (comparison group).

**Results:**

The intervention group showed a greater willingness to do the test (90% vs. 67%, *p* < 0.001) and to recommend it to others (96% vs. 74%, *p* < 0.001), ability to complete the test by themselves (94% vs. 56%, *p* < 0.001) and confidence in noticing symptoms (78% vs. 32%, *p* < 0.001) after the presentation compared to before. There was a significant difference between the intervention group post‐presentation and the comparison group on intention to do the test (90% vs. 79%, *p* = 0.02), recommending it to others (96% vs. 83%, *p* < 0.001), and confidence in their ability to complete the test by themselves (94% vs. 63%, *p* < 0.001).

**Conclusion:**

A culture‐sensitive, faith‐placed health education intervention delivered in mosques can substantially improve knowledge of bowel cancer screening and increase the intention to participate in the screening programme.

**Patient or Public Contribution:**

The intervention presentation was developed using insights from four public involvement sessions with four to six members representative of the East London Muslim community. The sessions sought attendees’ thoughts on appropriate ways to approach the intervention design for their community and asked for their views on the acceptability, appropriateness of messaging, format/design and likely impact of the presentation. Their views were then utilised to improve the presentation.

## 1 Introduction

Bowel cancer screening using testing for blood in faeces significantly reduces mortality from colorectal cancer [1, 2, 3]. In the United Kingdom, bowel cancer screening is currently offered to those aged 60–74 years, with an expansion to those aged 50–59 years underway [4]. Those registered with a GP are automatically sent an NHS bowel cancer screening kit every 2 years. Similarly, the US Preventive Services Task Force recommends that adults aged 45–75 be screened for colorectal cancer yearly [5]. In the United Kingdom, bowel cancer screening using faecal immunochemical testing (FIT) has the lowest participation rate of the three National Health Service (NHS) cancer screening programmes (breast, cervix and bowel) [6].

Participation rates are particularly low (< 40%) in deprived and inner‐city populations and in areas with substantial ethnic diversity, including East London [7]. Uptake has been shown to be lower particularly among South Asian populations compared to White British adults, and among Muslims compared to Christians [8]. In East London, a significant proportion of those from other ethnic groups are Muslim. For example, according to the UK Census, Muslims constitute 38% of the population of the borough of Tower Hamlets [9]. Because the introduction of bowel cancer screening with FIT has been observed to reduce the risk of dying from the disease by > 50% [10, 11], it is important to address the issue of low participation.

A number of interventions have been shown to be effective in increasing participation in bowel cancer screening, including postal and telephone reminders, primary care endorsements, repeat approaches to non‐participants and advance notifications [12]. Mosque‐placed interventions may provide a solution to increasing bowel screening rates among ethnic minorities who are Muslim. There are also particular religious beliefs that may affect how Muslim populations perceive bowel screening offers that could require tailored interventions to support their informed participation [13, 14]. This includes aligning the content of communications with the Muslim religion, as well as the setting, context and messenger by which these communications are delivered (so‐called faith‐placed interventions).

In support, qualitative studies on mosque‐based interventions to promote cancer screening participation in the United Kingdom and the United States suggest that such interventions are both feasible and acceptable [13, 14]. Faith‐placed interventions have shown some promise in increasing screening rates in other cancers such as breast cancer. For example, a mosque‐placed intervention in the United States was associated with a significant 29% increase in self‐reported likelihood of participation in breast cancer screening, and 38% of women who had not participated in the previous 2 years went on to have a mammogram in the year following the intervention [14]. However, there has been no such examination of the potential effectiveness of a mosque‐based intervention to increase screening for bowel cancer.

There is a need for studies to examine the effect of faith‐based interventions to promote bowel cancer screening. We aimed to investigate the effect of a mosque‐placed health education intervention on promoting participation in bowel cancer screening within the Islamic community in East London, with quantitative results on the effect of the intervention on bowel cancer perceptions and screening intentions.

## 2 Methods

This is a non‐randomised comparative intervention evaluation with anonymous survey questionnaire endpoints.

### 2.1 Faith‐Placed Intervention: Development and Description

Our intervention was a faith‐placed presentation on bowel cancer screening delivered in person by clinicians of the Muslim religion at mosques in East London. Faith‐placed interventions have a spiritual basis and occur in organised religious settings, whereas faith‐based interventions also have a spiritual basis but do not necessarily take place in religious establishments [15].

The intervention was delivered in partnership with the British Islamic Medical Association (BIMA), a group of Muslim clinician volunteers in the United Kingdom with experience in community health promotion. Together with the research team, they adapted an existing cancer screening intervention using materials from Cancer Research UK. Previous community outreach work with these assets had been awarded a Public Health England commendation. Following initial adaptation, the first version was user‐tested and further developed using insights from four public involvement sessions with members representative of the Muslim community in East London (two sessions with men and two sessions with women). Each session included four to six individuals, was single‐gender, and was co‐facilitated by two (same‐gender) members of the research team (one of Muslim religion and one of different religion, but with greater experience in focus group facilitation). The sessions started by introducing the participation issues that the intervention aimed to address. It also sought attendees’ thoughts on appropriate ways to approach the intervention design for their community. Attendees were then shown the full presentation and asked for their views on the acceptability, appropriateness of messaging, format/design and likely impact or reactions. Written notes were taken during the sessions, and the presentation was revised to accommodate their suggestions in consultation with the wider team.

The final presentation (File S1) used PowerPoint slides to present facts about bowel cancer, including its incidence and mortality in the United Kingdom. It then moved on to addressing topics related to screening and diagnosis of bowel cancer, including typical symptoms, the definition and purpose of screening for bowel cancer and its importance. The presentation then covered potential motivational and practical barriers to participation, the screening test used, and the instructions for carrying out the home sampling. It concluded with a summary of the risks versus benefits of participating in the screening. Dealing with these issues in a one‐to‐one telephone intervention has been shown to lead to improved bowel cancer screening participation [7]. All of the presentation content was non‐religious, except for one slide that addressed preventive measures such as screening, being in line with religious teachings, which was reinforced by faith leaders in attendance.

The presentation was approximately 30 min in duration, depending on the style of the presenter and interaction with the audience during the presentation. There was additional time for questions following the presentation, and the venues scheduled 1 h for the event.

### 2.2 Intervention Setting and Delivery

Our intervention sites were mosques in East London identified by BIMA. These were pragmatically chosen based on convenience from sites that were willing to participate and had either local clinicians in their congregation or were willing to platform and endorse an external clinician.

The presentation was given during sessions in mosques in East London (referred to as the intervention group). Although randomisation at the mosque level was considered, this was not possible due to the strong preference of mosques to be included in the intervention group. Scheduling was led by the mosques and guided by their knowledge of the attendance habits of their congregation. For this reason, some mosques chose to schedule the sessions close to congregational prayer times or study groups, whereas others held the sessions as separate events.

Recruitment of participants was through convenience, aiming to represent routine attendance. Participants were encouraged to specifically attend the sessions through regular communication channels such as posters, social media and announcements after routine mosque activities. Immediately before the sessions, faith leaders encouraged the attendance of those who were in the mosque, such as after congregational prayers or after study circles. All sessions were gender‐specific (either female only or male only). Presenters were clinicians who were either known and trusted in their community or local clinicians who were co‐platformed by trusted religious leaders. All but one presenter were Muslim. All activities were co‐endorsed with the mosque, BIMA and the regional NHS cancer alliance. Advance publicity for the session was through the mosques' usual outreach, and that of BIMA and the cancer alliance. The intervention was delivered between March and September 2022.

### 2.3 Participants

No exclusion criteria were applied to attendees, but those who were in the eligible age range for bowel cancer screening were actively encouraged to attend, with timings of sessions targeted to maximise participation in this age cohort. The presentation was delivered in English and translated into Bangla or Urdu either by the clinician themselves who was bilingual or by a mosque volunteer, as per the instruction of the mosque. Following the sessions, assistance was provided to those who wished to request a FIT test kit.

### 2.4 Measures

Participants were asked only for their age group to help protect individual identities (principle of data minimisation). Although we intended to collect individual‐level sex data (as each session was single‐sex), questionnaires from different sessions were mixed on collection at each mosque, making it impossible to do so. No other participant‐identifiable data were collected.

We administered a short survey of attitudes and perceptions of bowel cancer, and intentions to take part in bowel cancer screening, before and immediately after the presentation (Table 1). In addition, we administered a survey to a single male session in a further East London mosque where no presentation took place (referred to as the comparison group). Both of these surveys were developed specifically for this evaluation. Two of the questions (on having heard of the bowel cancer screening programme before and confidence that the subject would recognise symptoms) were based on similar questions found in the Bowel Cancer Awareness Measure [16]. Surveys were completed on paper in written English, and volunteers were on hand to help with translation and to help fill in the response options. Each participant was allocated a number which was linked to their pre‐intervention and post‐intervention questionnaire. This enabled the linking of the pre‐intervention with post‐intervention results without explicitly identifying the participants.

**Table 1:** Questions in the three surveys (pre‐intervention, post‐intervention and comparison group).

Question	Included in pre‐presentation questionnaire	Included in post‐presentation questionnaire	Included in comparison questionnaire
Have you heard of bowel cancer screening before today?	Y	N	Y
Have you ever been invited to do a stool test for bowel cancer screening?	Y	N	Y
Have you ever done a stool test for bowel cancer screening?	Y	N	Y
Will you do the stool test for bowel cancer screening when you are next invited?	Y	Y	Y
Will you recommend the stool test for bowel cancer screening to others?	Y	Y	Y
Bowel cancer screening is only necessary if I have symptoms.	Y	Y	Y
If bowel cancer is found early by screening, what is the person's chance of surviving?	Y	Y	Y
If you were sent the stool test for bowel cancer screening, would you feel able to complete it by yourself?	Y	Y	Y
How confident are you that you would notice a bowel cancer symptom?	Y	Y	Y
How much of the bowel cancer screening talk did you listen to today?	N	Y	N
How much of what you heard during the bowel cancer screening talk did you understand?	N	Y	N
How much of what you heard during the bowel cancer screening talk did you find useful?	N	Y	N
How do you feel about your mosque giving talks about bowel cancer screening?	N	Y	N

### 2.5 Analysis

Responses to questions were compared between intervention and comparison groups using the *χ* ^2^ test. Responses pre‐ and post‐presentation were compared within the intervention group using the McNemar test [17].

## 3 Results

### 3.1 Sample Characteristics

There were 204 subjects participating in the surveys before and after the presentation, and 72 participants in the comparison survey. The presentation was given to seven male and five female sessions in seven mosques in East London (referred to as the intervention group). The comparison group consisted of male participants only.

In the intervention group, 47% were aged ≤ 55, 41% were aged 56–70 and 12% were aged > 70. In the comparison group, 41% were aged ≤ 55, 41% were aged 56–70 and 18% were aged > 70. Age distribution did not differ significantly between intervention and comparison groups (*p* = 0.3). Survey responses are shown in Table 2.

**Table 2:** Survey responses and significance of differences between intervention and comparison and between pre‐presentation and post‐presentation in the intervention mosques.

		Intervention group pre‐presentation (*n* = 204)	Intervention group post‐presentation (*n* = 204)	Comparison (*n* = 71)	Significance pre‐presentation vs. comparison	Significance post‐presentation vs. comparison	Significance pre‐presentation vs. post‐presentation
Factor	Category	*N* (%)	*N* (%)	*N* (%)			
Age group	≤ 55	96 (47)	96 (47)	29 (41)	*p* = 0.3	NA	NA
	56–70	84 (41)	84 (41)	29 (41)			
	> 70	24 (12)	24 (12)	13 (18)			
Heard of bowel cancer screening?	Yes	92 (46)	NA	44 (61)	*p* = 0.03	NA	NA
	No/not sure	107 (54)	NA	28 (39)			
Ever invited?	Yes	55 (27)	NA	30 (42)	*p* = 0.1	NA	NA
	No/not sure	147 (73)	NA	42 (58)			
Ever done a test?	Yes	53 (27)	NA	26 (36)	*p* = 0.4	NA	NA
	No/not sure	146 (73)	NA	46 (64)			
Will you do test?	Yes^a^	131 (66)	179 (90)	56 (79)	*p* = 0.05	*p* = 0.02	*p* < 0.001
	No/don't know^a^	66 (34)	21 (10)	15 (21)			
Will you recommend test?	Yes^a^	146 (73)	194 (96)	59 (83)	*p* = 0.1	*p* < 0.001	*p* < 0.001
	No/don't know^a^	53 (27)	8 (4)	12 (17)			
Bowel cancer screening only needed for symptoms	Agree	70 (35)	54 (27)	26 (36)	*p* = 0.4	*p* < 0.001	*p* = 0.05
	Disagree	65 (33)	13 (68)	28 (39)			
	Don't know	65 (33)	10 (5)	18 (25)			
If bowel cancer is found early by screening, chance of survival?	Good	105 (52)	170 (85)	48 (67)	*p* = 0.03	*p* = 0.004	*p* < 0.001
	Fair	36 (18)	20 (10)	11 (15)			
	Poor	3 (1)	3 (1)	3 (4)			
	Not sure	58 (29)	8 (4)	10 (14)			
Could you complete test by yourself?	Yes^a^	115 (56)	188 (94)	45 (63)	*p* = 0.5	*p* < 0.001	*p* < 0.001
	No/don't know^a^	85 (44)	13 (6)	27 (37)			
How confident are you that you would notice bowel cancer symptoms?	Not at all confident	59 (29)	6 (3)	22 (31)	*p* = 0.08	*p* < 0.001	*p* < 0.001
	Not very confident	78 (39)	38 (19)	27 (37)			
	Fairly confident	47 (23)	80 (41)	10 (14)			
	Very confident	17 (9)	72 (37)	13 (18)			
How much of talk did you listen to?	All/most	NA	188 (94)	NA	NA	NA	NA
	Some	NA	8 (4)	NA			
	None	NA	3 (2)	NA			
How much did you understand?	All/most	NA	183 (91)	NA	NA	NA	NA
	Some	NA	16 (8)	NA			
	None	NA	2 (1)	NA			
How much did you find useful?	All	NA	127 (63)	NA	NA	NA	NA
	Most	NA	65 (32)	NA			
	Some	NA	8 (4)	NA			
	None	NA	3 (1)	NA			
How do you feel about mosque giving talks on bowel screening?	Good idea	NA	187 (93)	NA	NA	NA	NA
	Bad idea	NA	7 (3)	NA			
	Don't mind	NA	8 (4)	NA			

^a^ Yes = probably or definitely yes; No = probably or definitely no.

### 3.2 Pre‐Presentation Intervention Group Versus Comparison Group

Before the presentation, there were no significant differences between the intervention group and the comparison group in terms of having previously been invited to complete a test (*p* = 0.1), having ever performed the test (*p* = 0.4), willingness to recommend the test to others (*p* = 0.1), knowledge that screening is not for management of symptoms (*p* = 0.4), ability to complete the test unaided (*p* = 0.4) and confidence that the subject would notice symptoms of bowel cancer (*p* = 0.08). Before the presentation, the intervention group showed significantly lower awareness than the comparison group of the survival advantage of early detection (*p* = 0.03), with 52% being aware of good survival due to early stage diagnosis compared to 67% in the comparison group. Also before the intervention, the intervention group were significantly less likely than the comparison group to report having heard of bowel cancer screening (46% vs. 61%, *p* = 0.03). There was a borderline significant difference between pre‐presentation and comparison intention to complete the test (*p* = 0.05), with 66% intending to do so in the intervention group and 79% in the comparison group.

Age‐specific results are shown in Tables 3 (age ≤ 55) and 4 (age ≥ 56). The difference with respect to having heard of bowel cancer screening was confined to the subjects aged more than 56 (49% vs. 72%, *p* = 0.01). There was also a difference in this age group with respect to confidence in noticing symptoms (33% vs. 47%, *p* = 0.04). The differences in terms of awareness of good survival with early detection and intention to perform the test were unequivocally non‐significant within each age group.

**Table 3:** Survey responses and significance of differences between intervention and comparison and between pre‐presentation and post‐presentation in the intervention mosques for those aged 55 or under.

Factor	Category	Intervention group pre‐presentation	Intervention group post‐presentation	Comparison	Significance pre‐presentation vs. comparison	Significance post‐presentation vs. comparison	Significance pre‐presentation vs. post‐presentation
		*N* (%)	*N* (%)	*N* (%)			
Heard of bowel cancer screening?	Yes	41 (43)	NA	13 (45)	0.9	NA	NA
	No/not sure	54 (57)	NA	16 (55)			
Ever invited?	Yes	8 (8)	NA	5 (17)	0.2	NA	NA
	No/not sure	88 (92)	NA	24 (83)			
Ever done a test?	Yes	8 (9)	NA	5 (17)	0.4	NA	NA
	No/not sure	85 (91)	NA	24 (83)			
Will you do test?	Yes	56 (59)	85 (89)	26 (90)	0.002	> 0.95	< 0.001
	No/don't know	39 (41)	10 (11)	3 (10)			
Will you recommend test?	Yes	75 (79)	92 (97)	26 (90)	0.2	0.1	< 0.001
	No/don't know	20 (21)	3 (3)	3 (10)			
Bowel cancer screening only needed for symptoms	Agree	30 (31)	21 (22)	7 (24)	0.1	0.2	< 0.001
	Disagree	36 (38)	68 (72)	17 (59)			
	Don't know	30 (31)	6 (6)	5 (17)			
If bowel cancer found early by screening, chance of survival?	Good	47 (49)	79 (82)	19 (66)	0.1	0.09	< 0.001
	Fair	17 (18)	13 (14)	5 (17)			
	Poor	2 (2)	1 (1)	2 (7)			
	Not sure	29 (31)	3 (3)	3 (10)			
Could you complete test by yourself?	Yes	55 (59)	91 (95)	21 (72)	0.2	0.001	< 0.001
	No/don't know	39 (41)	5 (5)	8 (28)			
How confident are you that you would notice bowel cancer symptoms?	Not at all confident	33 (35)	2 (2)	13 (45)	0.2	< 0.001	< 0.001
	Not very confident	33 (35)	18 (20)	13(45)			
	Fairly confident	22 (23)	39 (43)	2 (7)			
	Very confident	7 (7)	32 (35)	1 (3)			
How much of talk did you listen to?	All/most	NA	88 (93)	NA	NA	NA	NA
	Some	NA	4 (4)	NA			
	None	NA	3 (3)	NA			
How much did you understand?	All/most	NA	88 (92)	NA	NA	NA	NA
	Some	NA	6 (6)	NA			
	None	NA	2 (2)	NA			
How much did you find useful?	All	NA	66 (69)	NA	NA	NA	NA
	Most	NA	25 (26)	NA			
	Some	NA	3 (3)	NA			
	None	NA	2 (2)	NA			
How do you feel about mosque giving talks on bowel screening?	Good idea	NA	84 (90)	NA	NA	NA	NA
	Bad idea	NA	5 (5)	NA			
	Don't mind	NA	5 (5)	NA			

### 3.3 Post‐Presentation Intervention Group Versus Comparison Group

The intervention group after presentation on all counts gave significantly different responses to the comparison group. After the presentation, 90% of intervention participants said they would do the test compared to only 79% in the comparison group (*p* = 0.02). Additionally, 96% of intervention participants were willing to recommend the test compared to 83% of the comparison group (*p* < 0.001). Furthermore, 94% of post‐presentation participants felt able to complete the test by themselves, compared to 63% in the comparison group (*p* < 0.001).

As for knowledge regarding cancer screening the differences between the comparison group and the post‐presentation intervention group were significant. Whereas 68% of the post group believed that cancer screening is necessary even if you do not display symptoms, only 39% of the comparison group thought this (*p* < 0.001). People's estimations of survivability if the disease is diagnosed early differed significantly, with 95% of the post‐presentation intervention group estimating their chances as good or fair compared to 82% of the comparison group (*p* = 0.004). After the presentation, 78% of intervention participants felt fairly or very confident that they would notice symptoms, whereas 32% of the comparison group felt the same (*p* < 0.001).

The significant differences were mainly due to the age group of > 55 years (Table 4). However, the younger age group also showed significant differences in confidence in completion of the test unaided (*p* = 0.001) and confidence in noticing symptoms (*p* < 0.001).

**Table 4:** Survey responses and significance of differences between intervention and comparison and between pre‐presentation and post‐presentation in the intervention mosques for those aged 56 or over.

		Intervention group pre‐presentation	Intervention group post‐presentation	Comparison	Significance pre‐presentation vs. comparison	Significance post‐presentation vs. comparison	Significance pre‐presentation vs. post‐presentation
Factor	Category	*N* (%)	*N* (%)	*N* (%)			
Heard of bowel cancer screening?	Yes	51 (49)	NA	31 (72)	0.01	NA	NA
	No/not sure	53 (51)	NA	12 (28)			
Ever invited?	Yes	47 (44)	NA	25 (58)	0.2	NA	NA
	No/not sure	59 (56)	NA	18 (42)			
Ever done a test?	Yes	45 (42)	NA	21 (49)	0.5	NA	NA
	No/not sure	61 (58)	NA	22 (51)			
Will you do test?	Yes	75 (74)	94 (90)	30 (71)	0.8	0.006	0.003
	No/don't know	27 (26)	11 (10)	12 (29)			
Will you recommend test?	Yes	71 (68)	102 (95)	33 (79)	0.2	0.002	< 0.001
	No/don't know	33 (32)	5 (5)	9 (21)			
Bowel cancer screening only needed for symptoms	Agree	40 (38)	33 (31)	19 (44)	0.8	< 0.001	0.3
	Disagree	29 (28)	68 (65)	11 (26)			
	Don't know	35 (34)	4 (4)	13 (30)			
If bowel cancer found early by screening, chance of survival?	Good	58 (54)	91 (87)	29 (67)	0.4	0.04	< 0.001
	Fair	19 (18)	7 (7)	6 (14)			
	Poor	1 (1)	2 (2)	1 (2)			
	Not sure	29 (27)	5 (5)	7 (16)			
Could you complete test by yourself?	Yes	60 (57)	97 (92)	24 (56)	0.9	< 0.001	< 0.001
	No/don't know	46 (43)	8 (8)	19 (44)			
How confident are you that you would notice bowel cancer symptoms?	Not at all confident	26 (25)	4 (4)	9 (21)	0.04	0.001	< 0.001
	Not very confident	45 (42)	20 (19)	14 (33)			
	Fairly confident	25 (24)	41 (39)	8 (19)			
	Very confident	10 (9)	40 (38)	12 (28)			
How much of talk did you listen to?	All/most	NA	70 (67)	NA	NA	NA	NA
	Some	NA	30 (29)	NA			
	None	NA	4 (4)	NA			
How much did you understand?	All/most	NA	56 (53)	NA	NA	NA	NA
	Some	NA	39 (37)	NA			
	None	NA	10 (10)	NA			
How much did you find useful?	All	NA	61 (57)	NA	NA	NA	NA
	Most	NA	40 (37)	NA			
	Some	NA	5 (5)	NA			
	None	NA	1 (1)	NA			
How do you feel about mosque giving talks on bowel screening?	Good idea	NA	103 (95)	NA	NA	NA	NA

### 3.4 Pre‐Presentation Intervention Group Versus Post‐Presentation Intervention Group

The intervention group gave significantly different responses to the questions after being given the presentation compared to before. Before the presentation, only 66% of participants said they would do the test compared to 90% post‐presentation (*p* < 0.001). Additionally, 73% said they would recommend the test before the presentation compared to 96% post‐presentation (*p* < 0.001). After the presentation, 94% of participants felt able to complete the test by themselves, compared to 56% before (*p* < 0.001). After the presentation, the proportion of those who were fairly or very confident they could notice symptoms increased from 32% to 78% (*p* < 0.001). Only 33% of the intervention group before the presentation believed that cancer screening was necessary even with no symptoms, compared to 68% post‐presentation (*p* = 0.05). Estimations of survivability changed significantly, with 95% post‐presentation estimating survival as good or fair, compared to 70% before the presentation (*p* < 0.001).

The age‐specific results were very similar to the overall results (Tables 3 and 4).

## 4 Discussion

This is the first project to examine the quantitative effects of a faith‐placed health education intervention to promote participation in bowel cancer screening on bowel cancer perceptions and screening intentions in the Islamic community in East London. We have found that a faith‐placed intervention has significant and positive effects on knowledge of screening (its necessity and effect on survivorship), willingness to participate in screening, confidence in completing the test and recommending bowel cancer screening to others. It is worth noting that levels of awareness and screening intentions were lower in the intervention group compared to the comparison group before the intervention, but higher after the intervention.

### 4.1 Context of Findings With the Literature

Faith‐placed interventions offer multiple potential advantages that have been well described previously [15]. Faith venues allow us to reach a large audience, provide a platform for message delivery, offer immediate social support, and allow messages to be delivered or reinforced by trusted and influential faith leaders and medical professionals.

In their recent systematic review, Adbul Latip et al. [18] found 42 RCTs that have previously tested interventions to increase colorectal cancer screening uptake in ethnic minority populations. Of these, none took place in mosques or targeted Muslim populations. Twelve studies (28%) took place in churches targeting either Korean American, Filipino‐American or African‐American adults with improved screening rates among the intervention groups compared to control groups. Although this highlights that faith‐placed interventions can be effective, as participation rates among Muslims are lower than Christians, the challenges of increasing rates among Muslims are likely to be specific.

Previous qualitative work by Christie‐de Jong et al. [13] has indicated that mosque‐based interventions (delivered via Zoom rather than in a place of worship) to promote colorectal screening are acceptable to Scottish Muslim women. We know of only one other study that quantitatively assessed a mosque‐placed intervention to promote cancer screening. In a US study, Padela et al. [14] delivered a mosque‐based, peer‐led intervention involving facilitated discussions and expert‐led lectures conveying health‐related religious teachings, and information about the benefits and process of mammography to 58 women. They found a significant 29% increase in self‐reported likelihood of participation in breast cancer screening, and 38% of women who had not participated in the previous 2 years had a mammogram in the year post‐intervention. Here we present findings from a larger cohort of both men and women, demonstrating that a faith‐placed intervention is effective in increasing rates of intention to participate in colorectal screening in East London.

### 4.2 Strengths and Limitations

Firstly, we utilised a good sample size of adults compared to other studies in the literature, which increases the power of our results. We also present a robust evaluation design and theory‐based intervention. We were limited in the size of the comparison group due to the reluctance of mosques to participate without being offered interventions, and resource limitations did not permit us to offer time‐delayed interventions. Because we relied on convenience sampling and conducted sessions within the mosques in same‐sex groups, we were unable to control for sex. The only comparison group session we were able to book was a male one. This reluctance was due to sites being unwilling to participate without being offered the intervention. Given the potential for gender‐specific differences in perceptions about cancer and screening (e.g., in colonoscopy in Singaporean populations) [19], gender‐stratified analyses would be important in future studies. Finally, we were only able to collect age data by age group and were not able to control for this. The sample size was not large enough to power age‐stratified analyses.

The North East London Cancer Alliance assessed the project and had no ethical concerns. We utilised the NHS Health Research Authority (HRA) tool to determine whether we needed to proceed to Research Ethics Committee (REC) approval, and the result indicated we did not need approval. We would have liked to have offered the intervention post hoc to the control mosques, which may have also increased the agreement of mosques to participate as controls. We were unable to do this as we were constrained by resources, but this could be a possible future strategy.

## 5 Conclusions

Our findings are consistent with a positive effect of faith‐placed interventions on knowledge and the intention to participate in bowel cancer screening in mosques in East London. Given that inequality levels related to screening are high, our findings indicate that commissioners should consider how they might engage with faith communities, and fund and embed such interventions into existing programmes to try and tackle the burden such inequality generates. Such interventions have the potential to lead to increased bowel cancer screening uptake, increase earlier detection and therefore increase bowel cancer survivorship among ethnic minorities. Future research should consider testing this intervention within a randomised‐control trial design with a larger sample size to allow for stratified analyses across age and gender. Furthermore, it will be important for future studies to follow up to see if the intention to test translates into higher FIT testing rates.

## Author Contributions

**Salman Waqar:** conceptualisation, methodology, investigation, visualisation, writing–review and editing. **Dharani Yerrakalva:** writing–original draft, writing–review and editing. **Thomas E. Duffy:** formal analysis, writing–review and editing. **Jake Chambers:** methodology, conceptualisation, writing–review and editing, investigation. **Zohra Ali:** writing–review and editing, methodology, investigation, conceptualisation. **Paul Thomas:** conceptualisation, methodology, writing–review and editing. **Caroline Cook:** conceptualisation, methodology, writing–review and editing. **Sufia Alam:** conceptualisation, methodology, investigation, writing–review and editing. **Leena Khagram:** conceptualisation, methodology, writing–review and editing, investigation. **Samantha Quaife:** conceptualisation, methodology, investigation, writing–review and editing. **Stephen W. Duffy:** conceptualisation, methodology, supervision, writing–review and editing, formal analysis.

## Consent

The authors have nothing to report.

## Conflicts of Interest

The authors declare no conflicts of interest.

## Supporting information

Supporting information.

## Data Availability

Data may be made available with certain assurances and applications should be sent to Professor Stephen Duffy.
