# Supplementary material for: Evaluation of a Faith‐Placed Health Education Service on Bowel Cancer Screening in Mosques in East London
Source: Health Expect. 2024 Aug 24;27(4):e70006. doi: 10.1111/hex.70006 (PMC11344223; doi:10.1111/hex.70006)
Supplement: Supplementary file 1 — Supporting information. [file HEX-27-e70006-s001.pptx]

## Slide 1
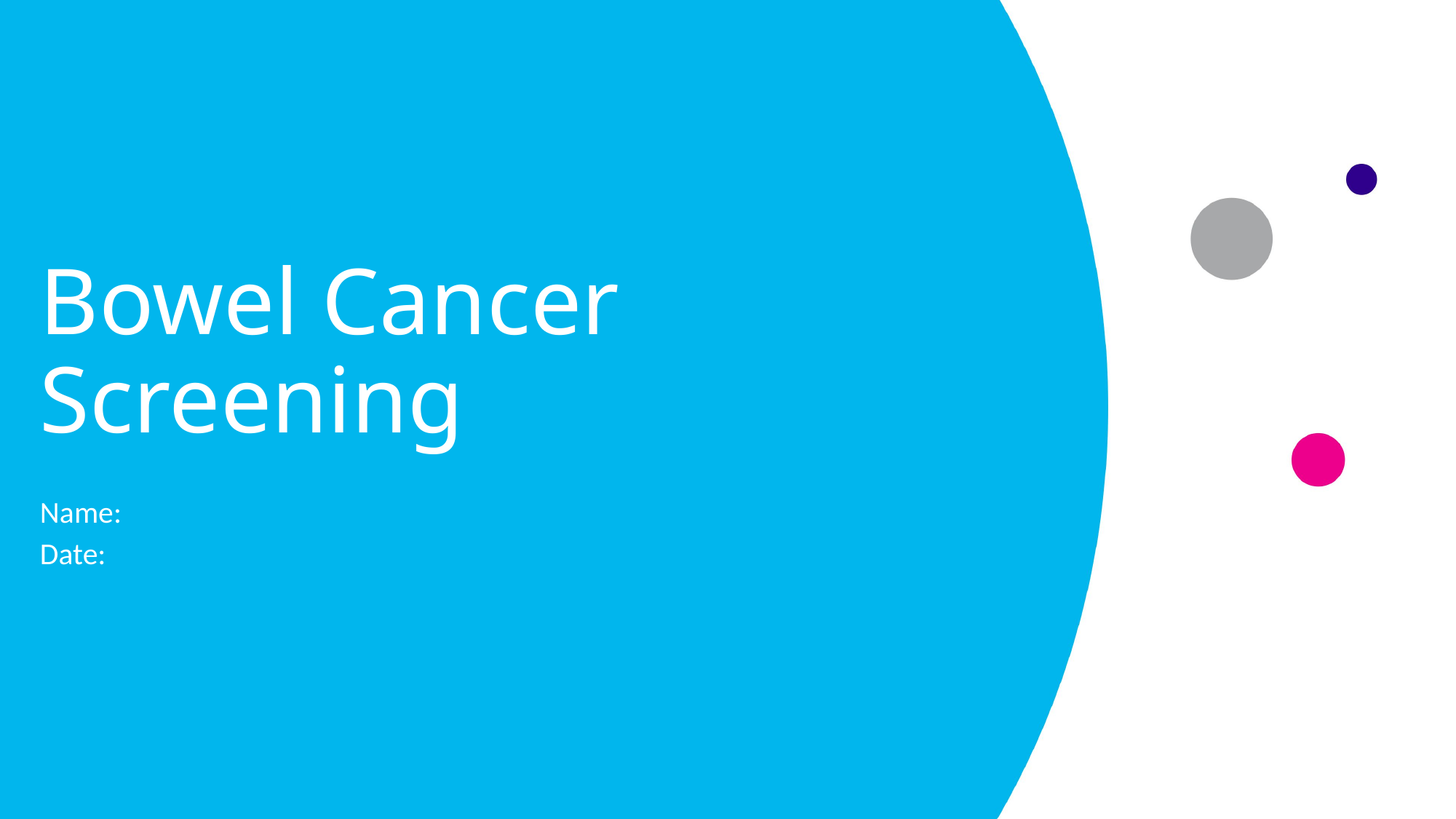

# Bowel Cancer Screening
Name:
Date:

## Slide 2
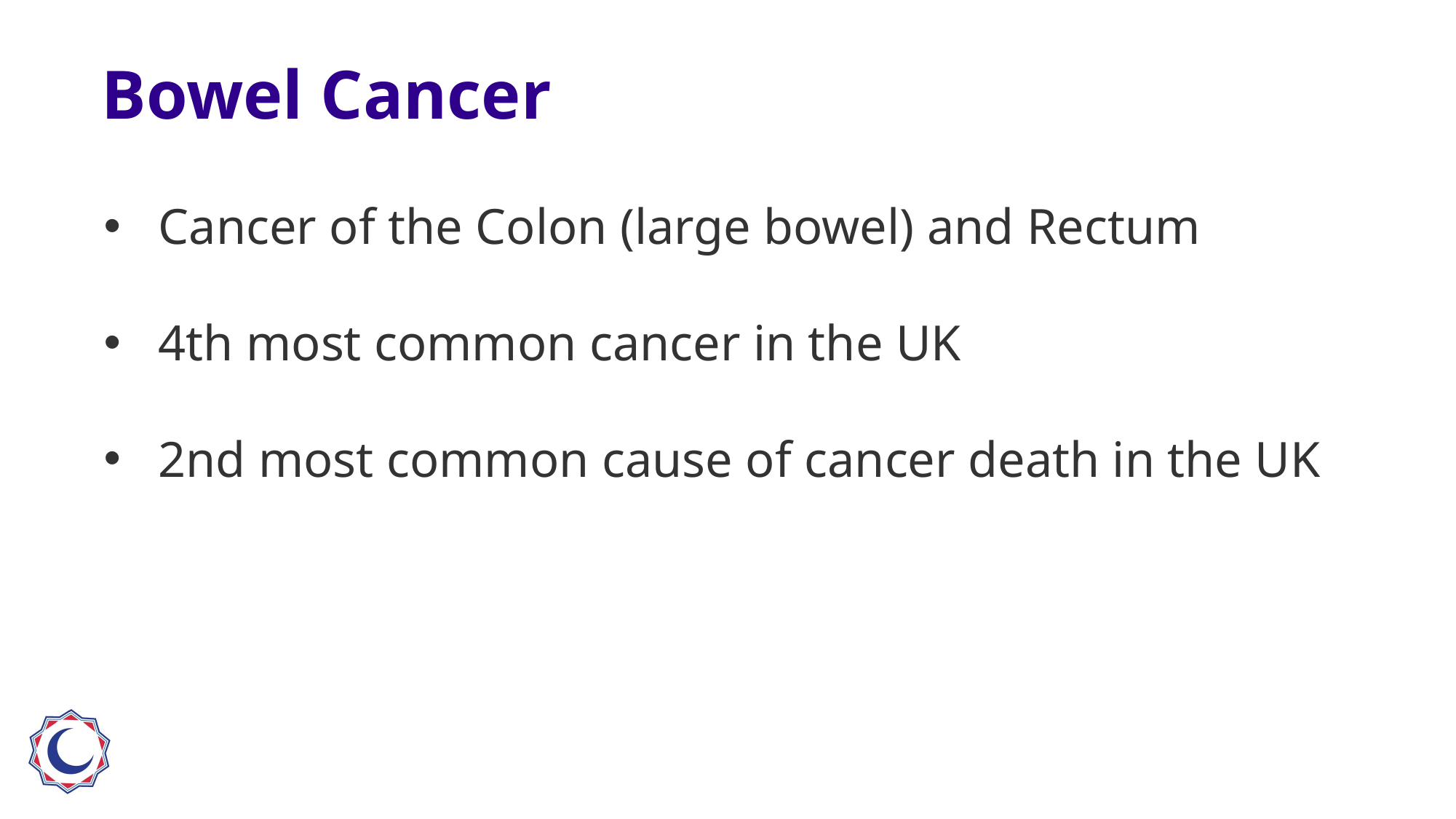

Bowel Cancer
Cancer of the Colon (large bowel) and Rectum
4th most common cancer in the UK
2nd most common cause of cancer death in the UK

## Slide 3
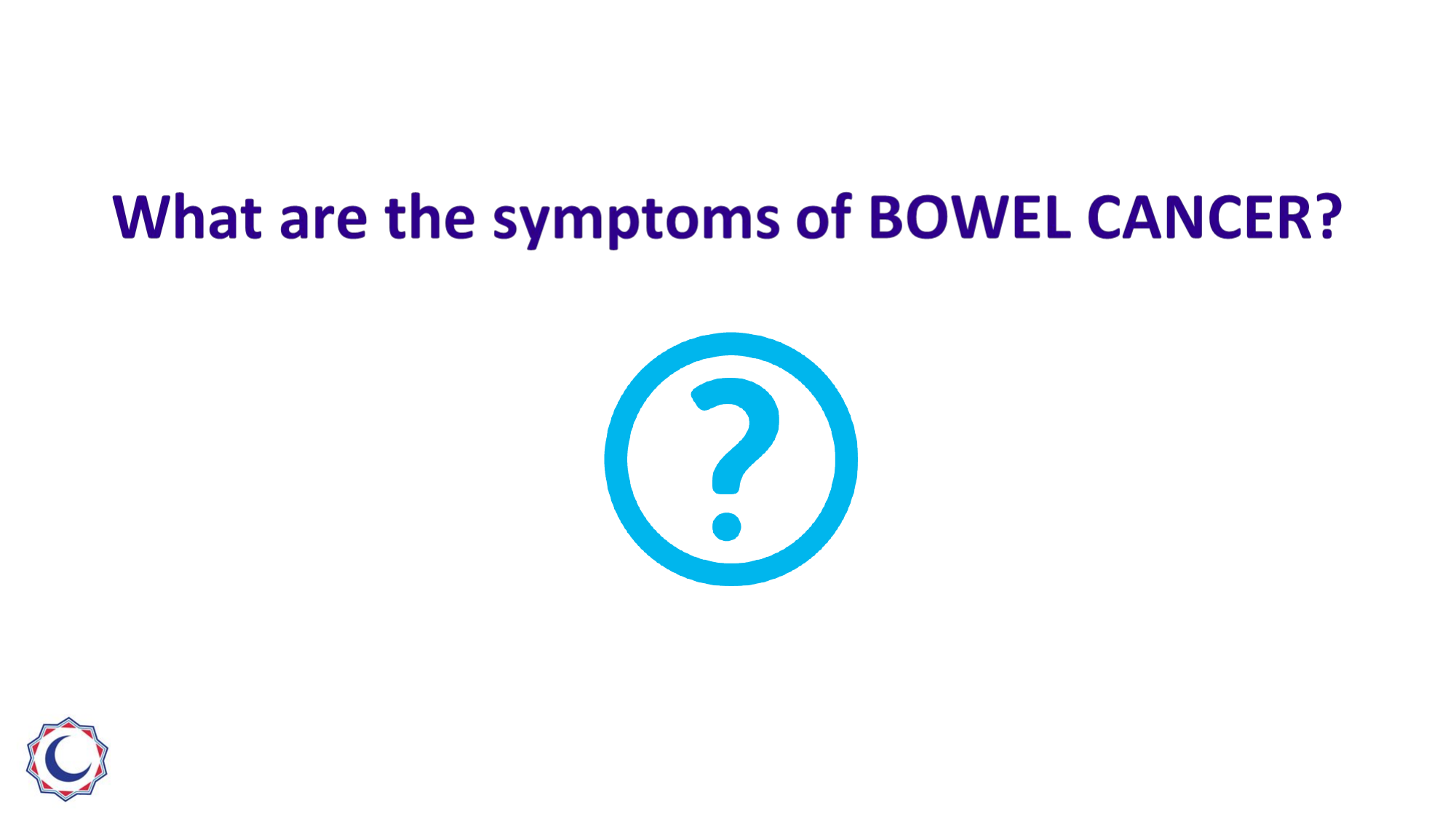

## Slide 4
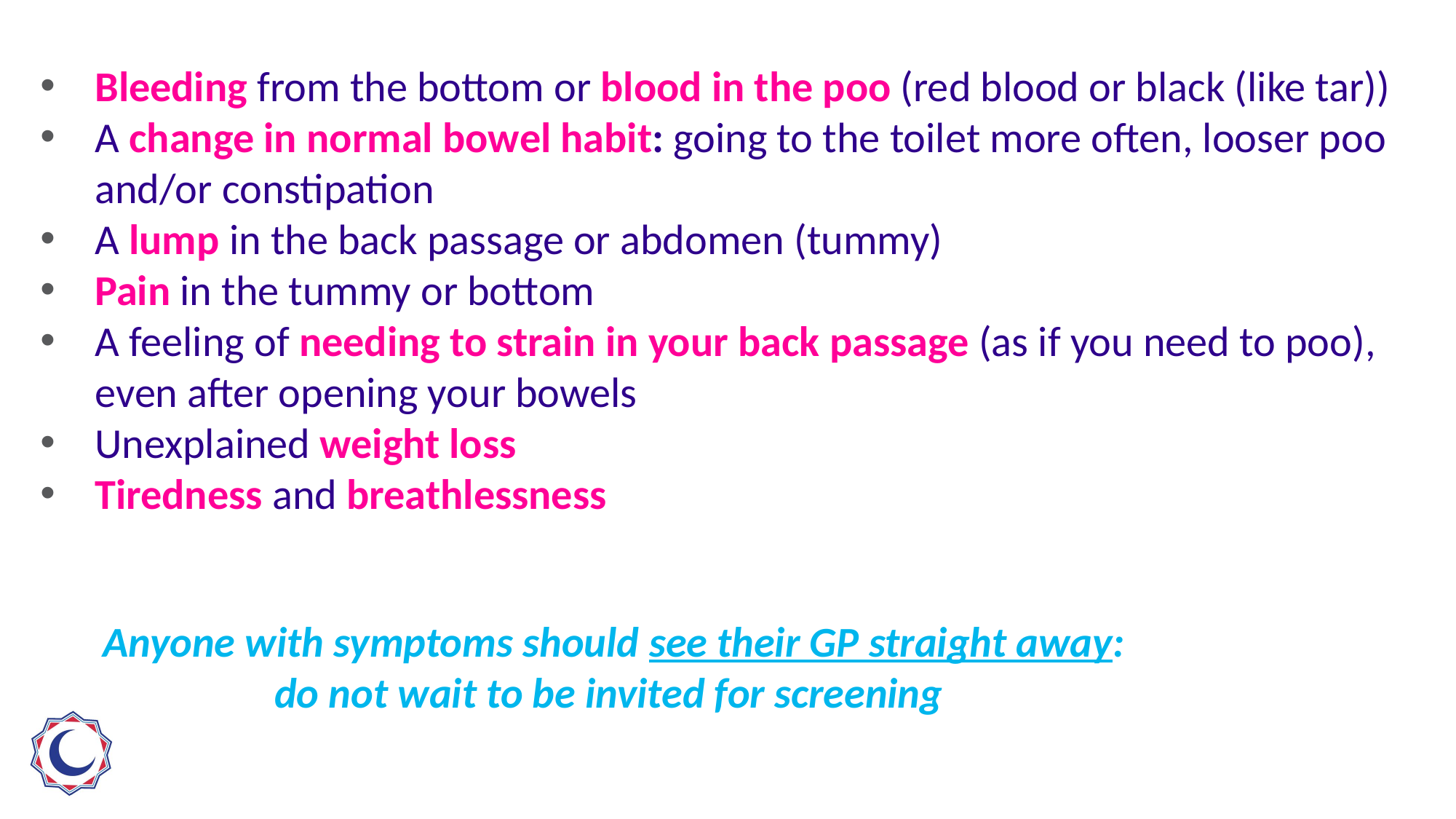

Bleeding from the bottom or blood in the poo (red blood or black (like tar))
A change in normal bowel habit: going to the toilet more often, looser poo and/or constipation
A lump in the back passage or abdomen (tummy)
Pain in the tummy or bottom
A feeling of needing to strain in your back passage (as if you need to poo), even after opening your bowels
Unexplained weight loss
Tiredness and breathlessness
Anyone with symptoms should see their GP straight away: do not wait to be invited for screening

## Slide 5
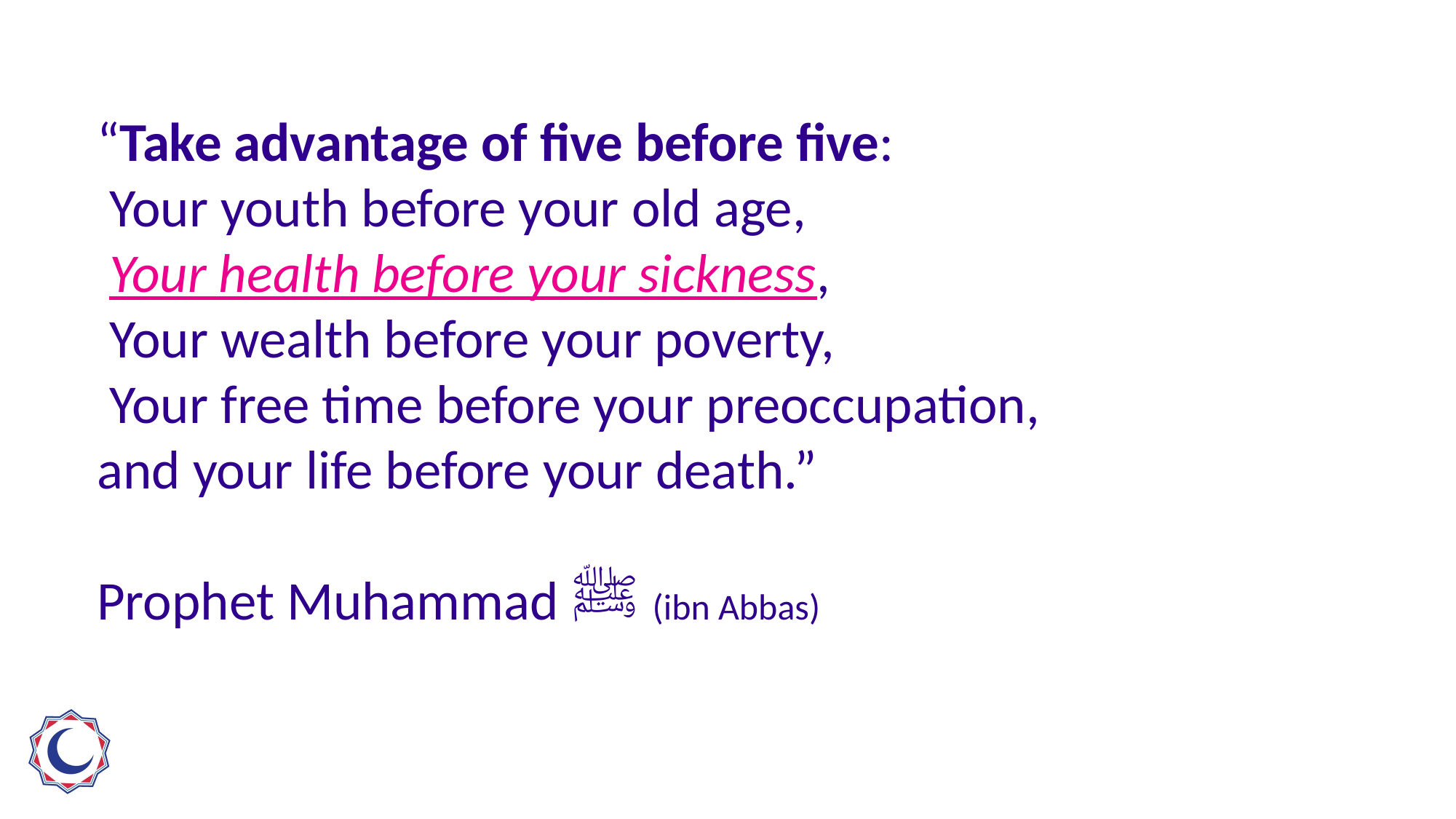

“Take advantage of five before five:
 Your youth before your old age,
 Your health before your sickness,
 Your wealth before your poverty,
 Your free time before your preoccupation,
and your life before your death.”
Prophet Muhammad ﷺ (ibn Abbas)

## Slide 6
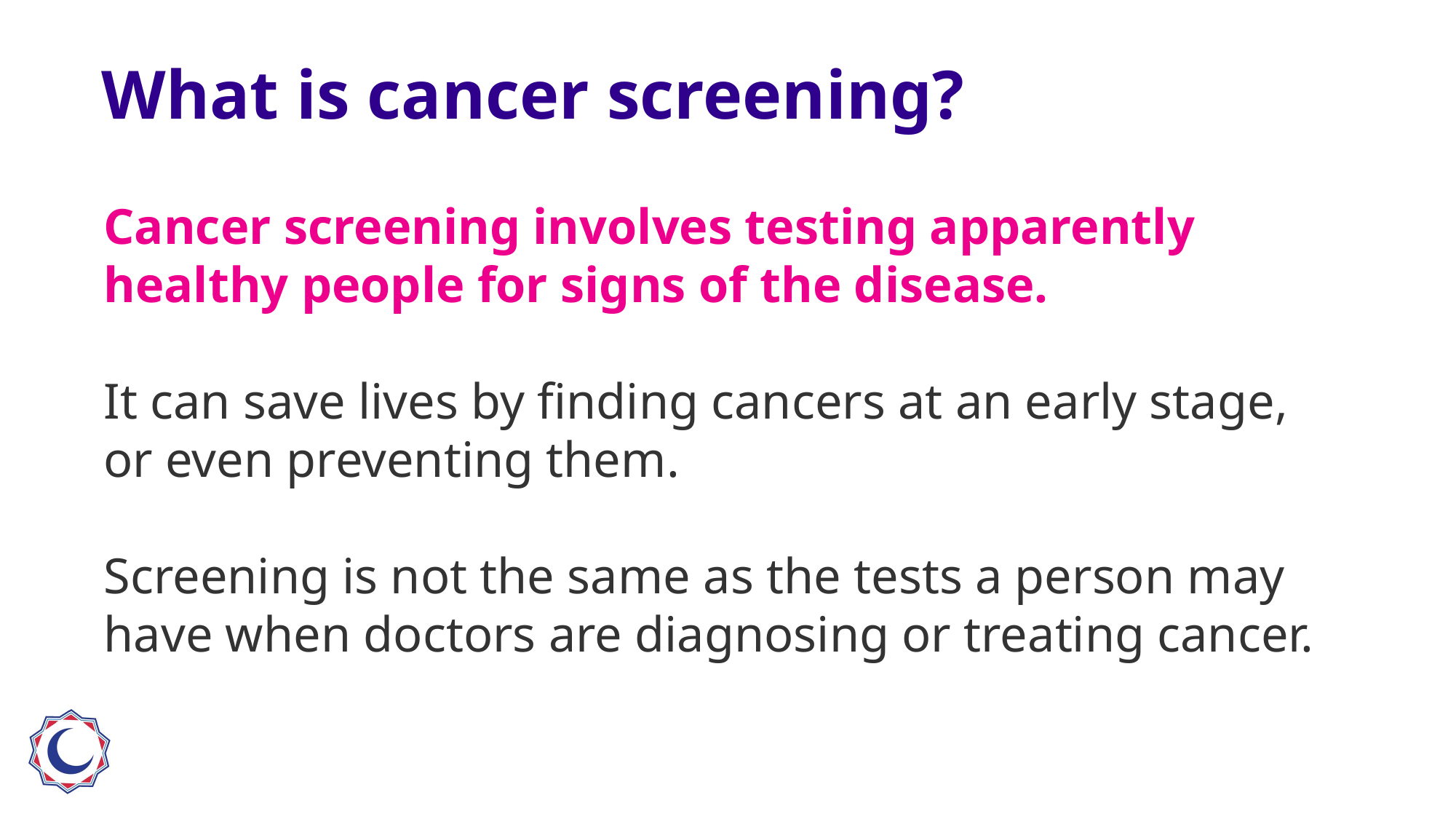

What is cancer screening?
Cancer screening involves testing apparently healthy people for signs of the disease.
It can save lives by finding cancers at an early stage, or even preventing them.
Screening is not the same as the tests a person may have when doctors are diagnosing or treating cancer.

## Slide 7
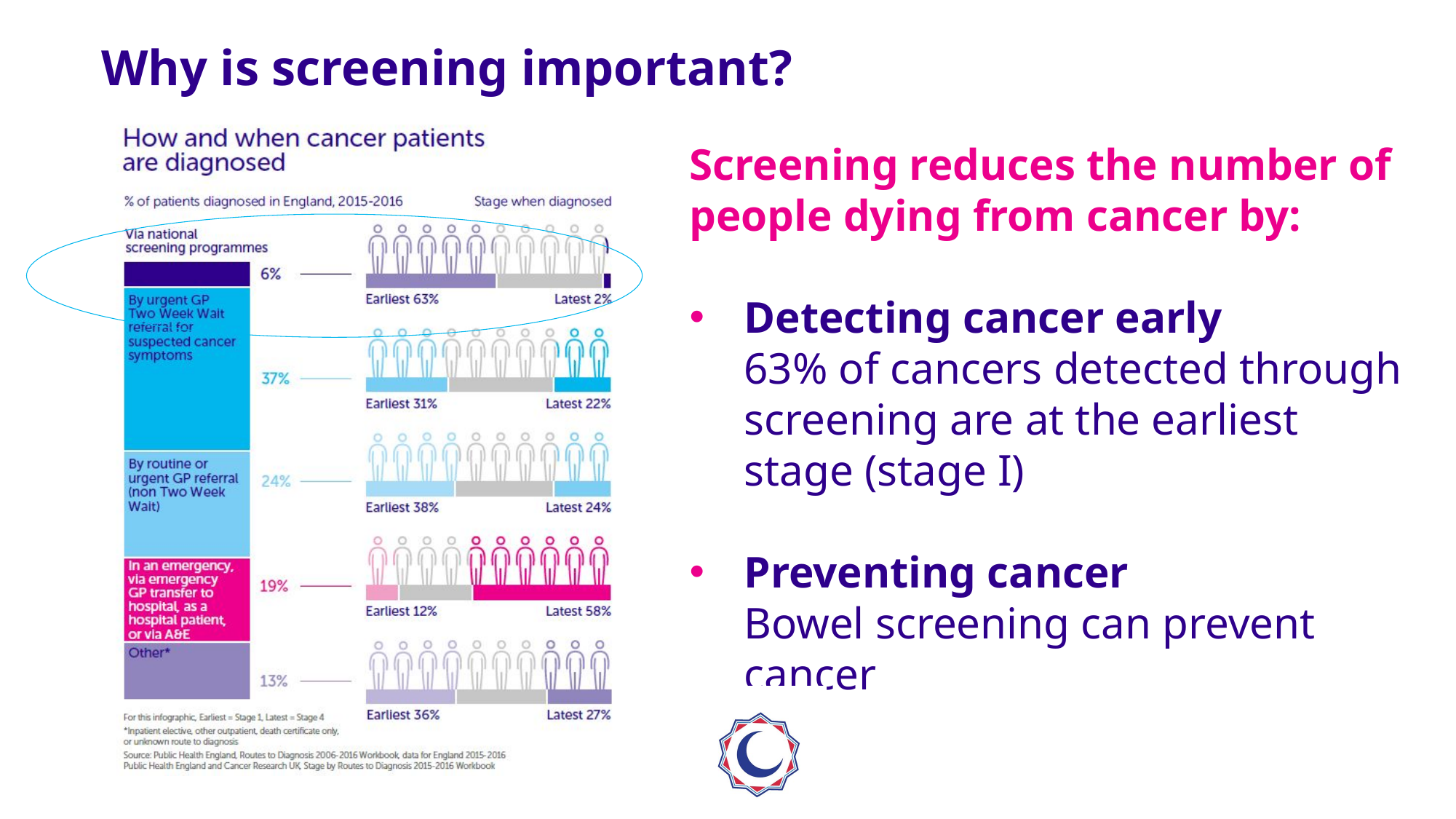

Why is screening important?
Screening reduces the number of people dying from cancer by:
Detecting cancer early
63% of cancers detected through screening are at the earliest stage (stage I)
Preventing cancer
Bowel screening can prevent cancer

## Slide 8
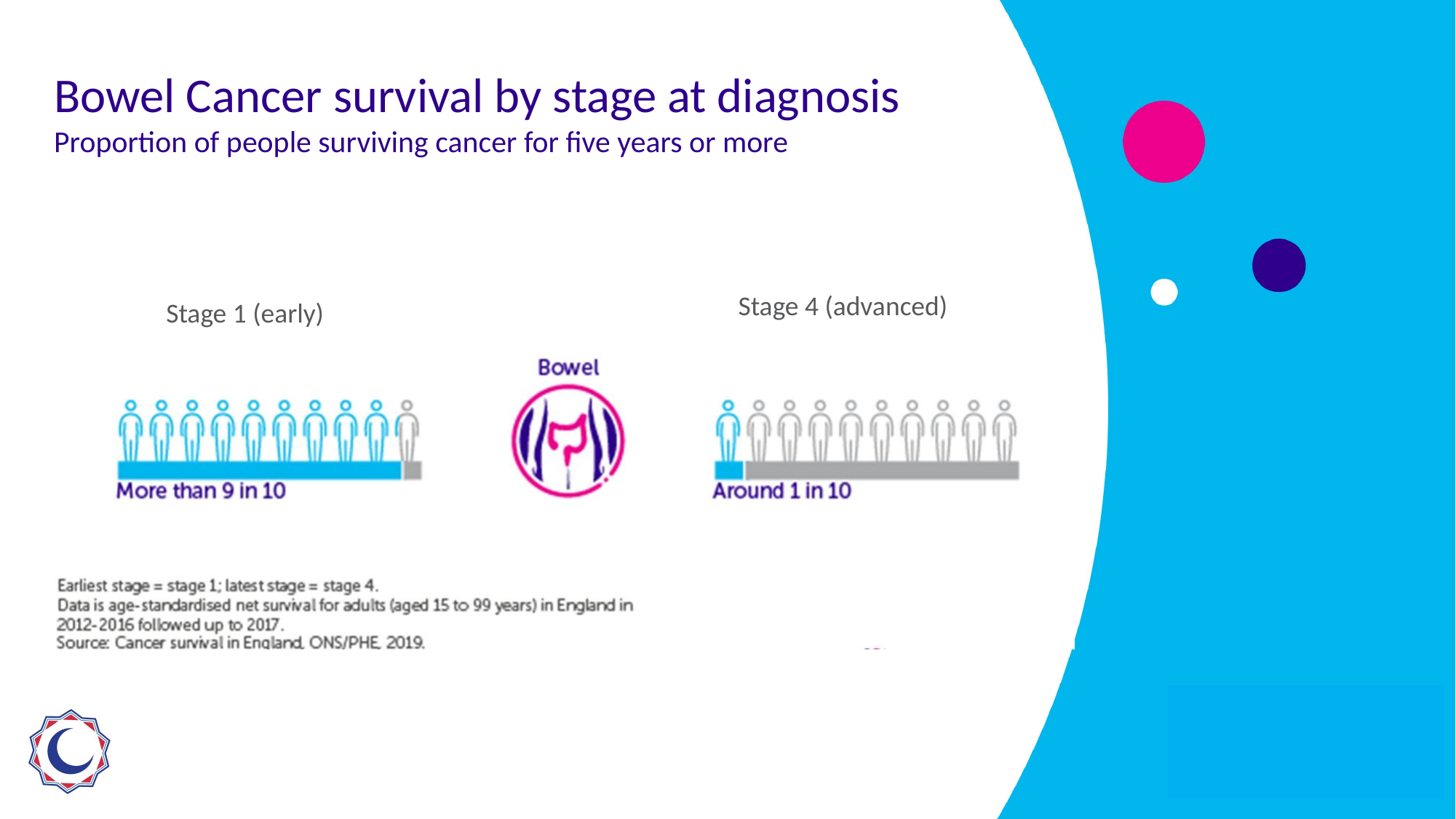

Bowel Cancer survival by stage at diagnosis
Proportion of people surviving cancer for five years or more
Stage 4 (advanced)
Stage 1 (early)

## Slide 9
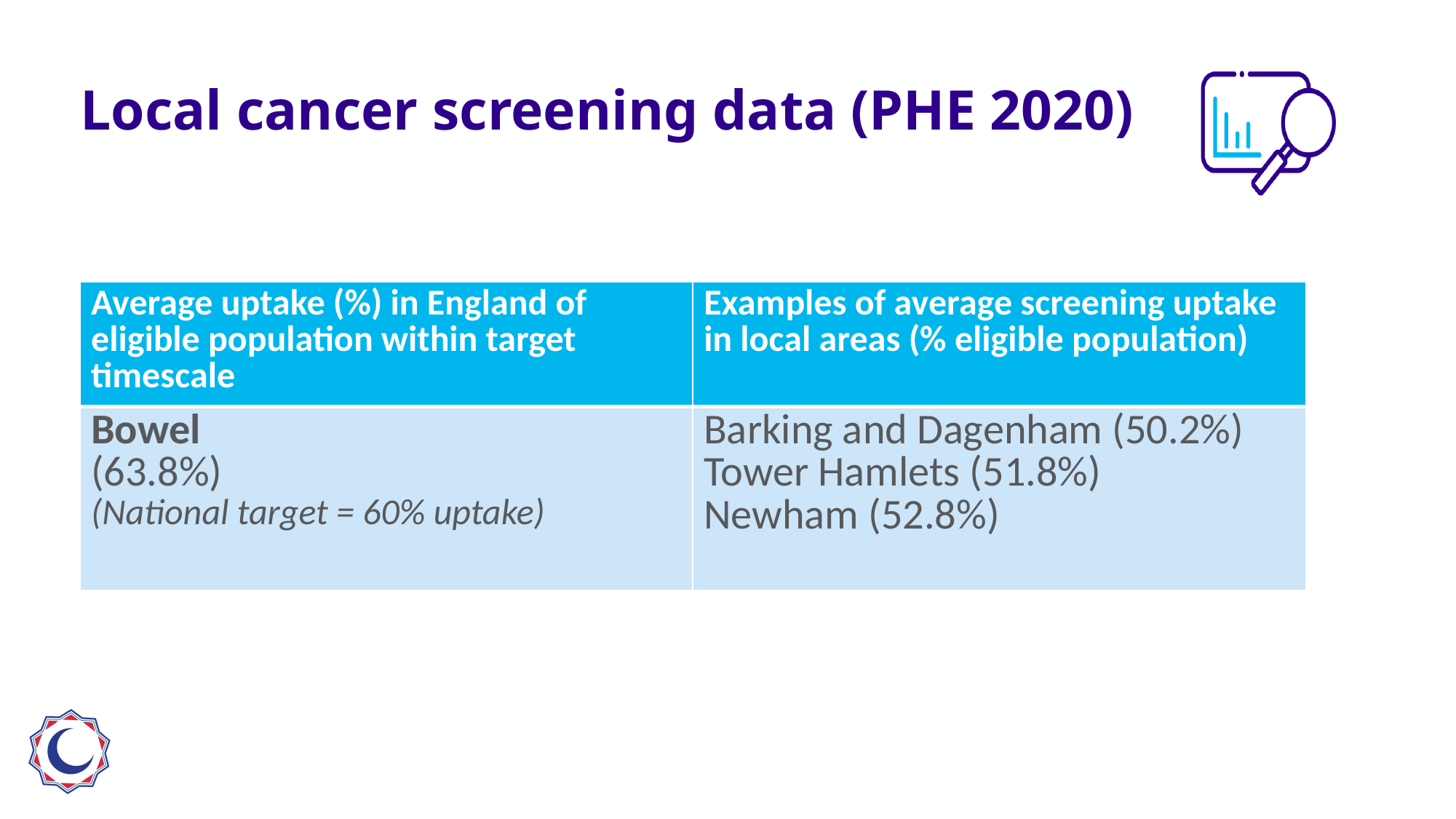

# Local cancer screening data (PHE 2020):
| Average uptake (%) in England of eligible population within target timescale | Examples of average screening uptake in local areas (% eligible population) |
| --- | --- |
| Bowel (63.8%) (National target = 60% uptake) | Barking and Dagenham (50.2%) Tower Hamlets (51.8%) Newham (52.8%) |

## Slide 10
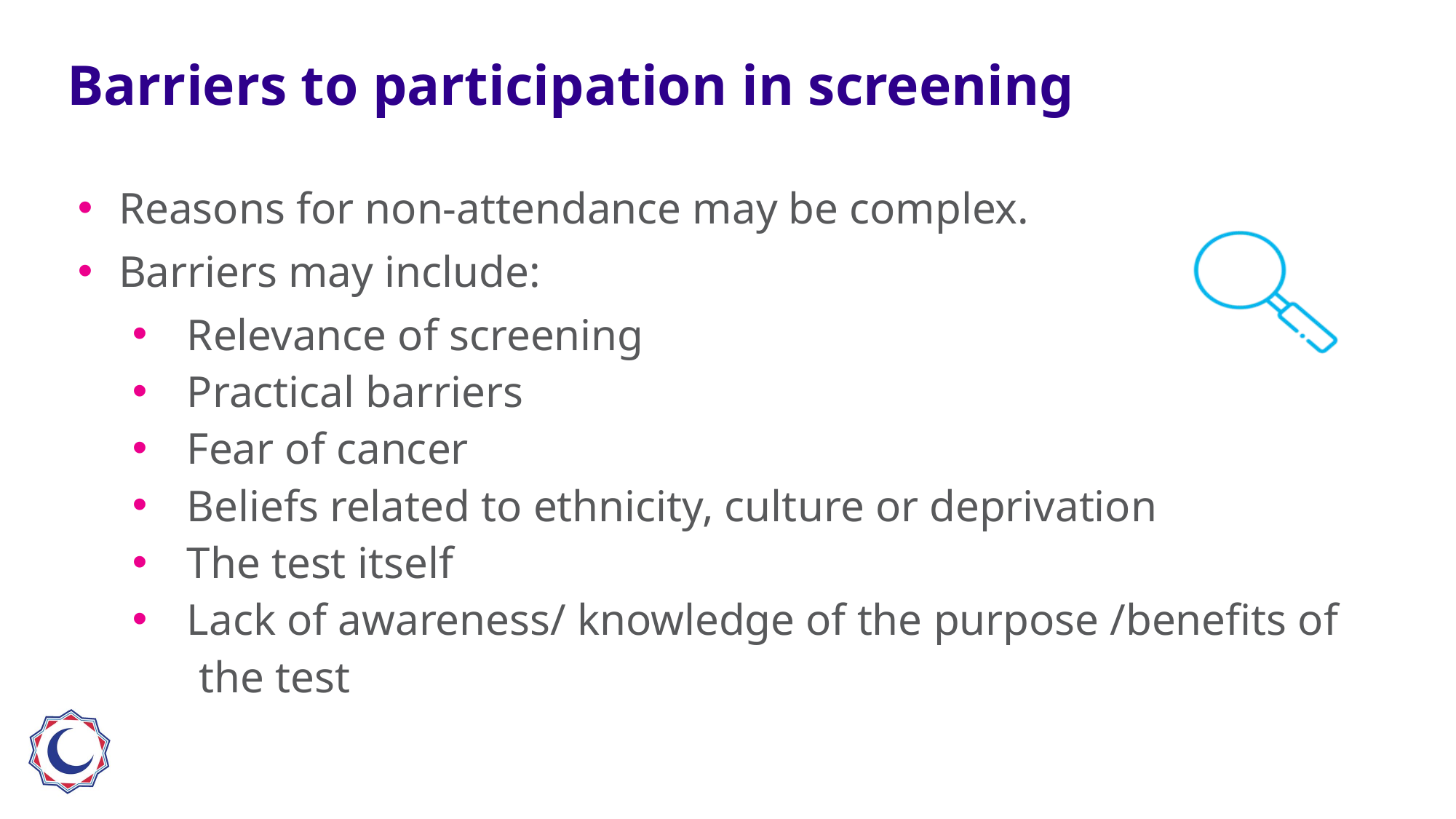

Barriers to participation in screening
Reasons for non-attendance may be complex.
Barriers may include:
Relevance of screening
Practical barriers
Fear of cancer
Beliefs related to ethnicity, culture or deprivation
The test itself
Lack of awareness/ knowledge of the purpose /benefits of
 the test

## Slide 11
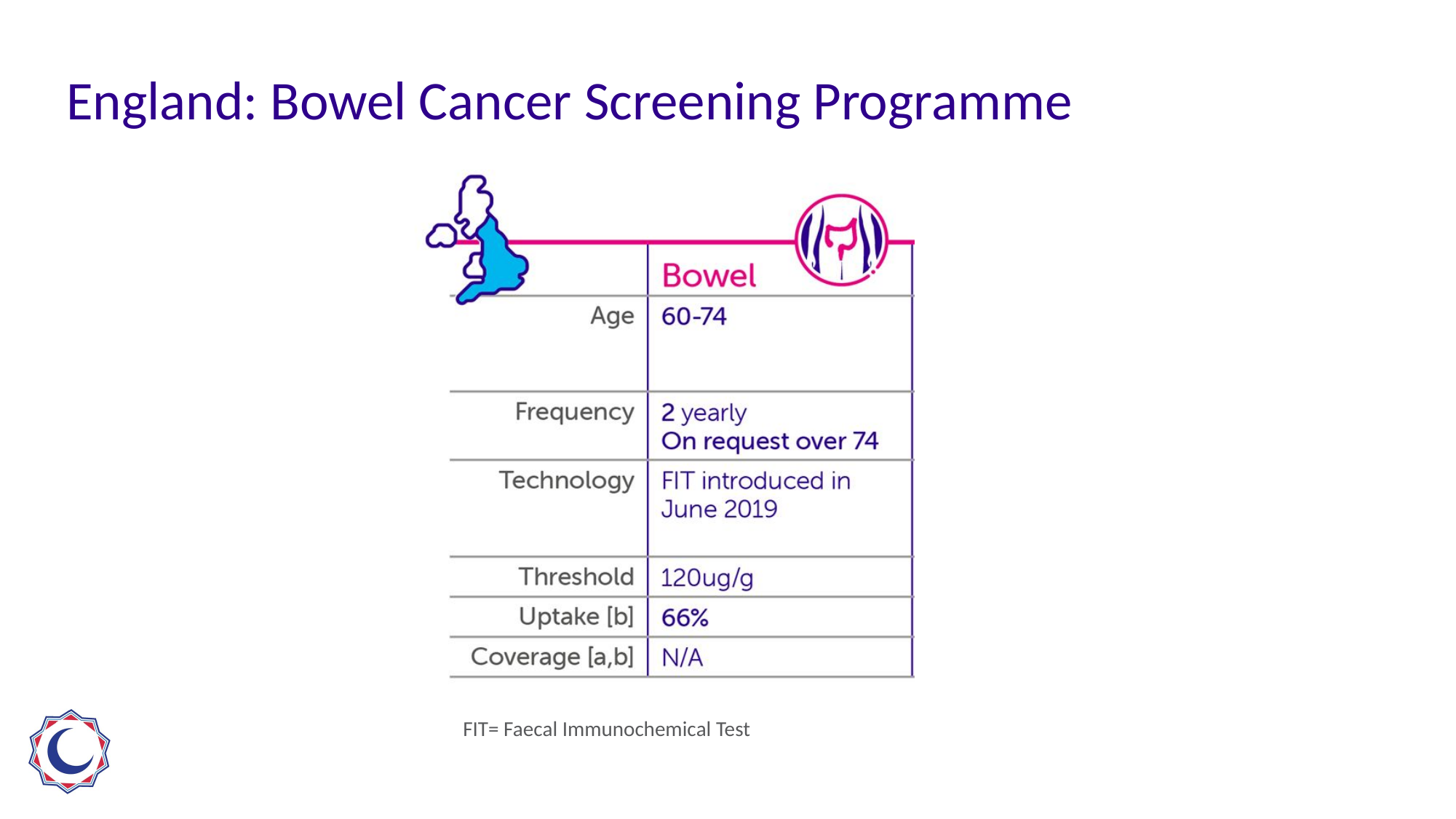

England: Bowel Cancer Screening Programme
FIT= Faecal Immunochemical Test

## Slide 12
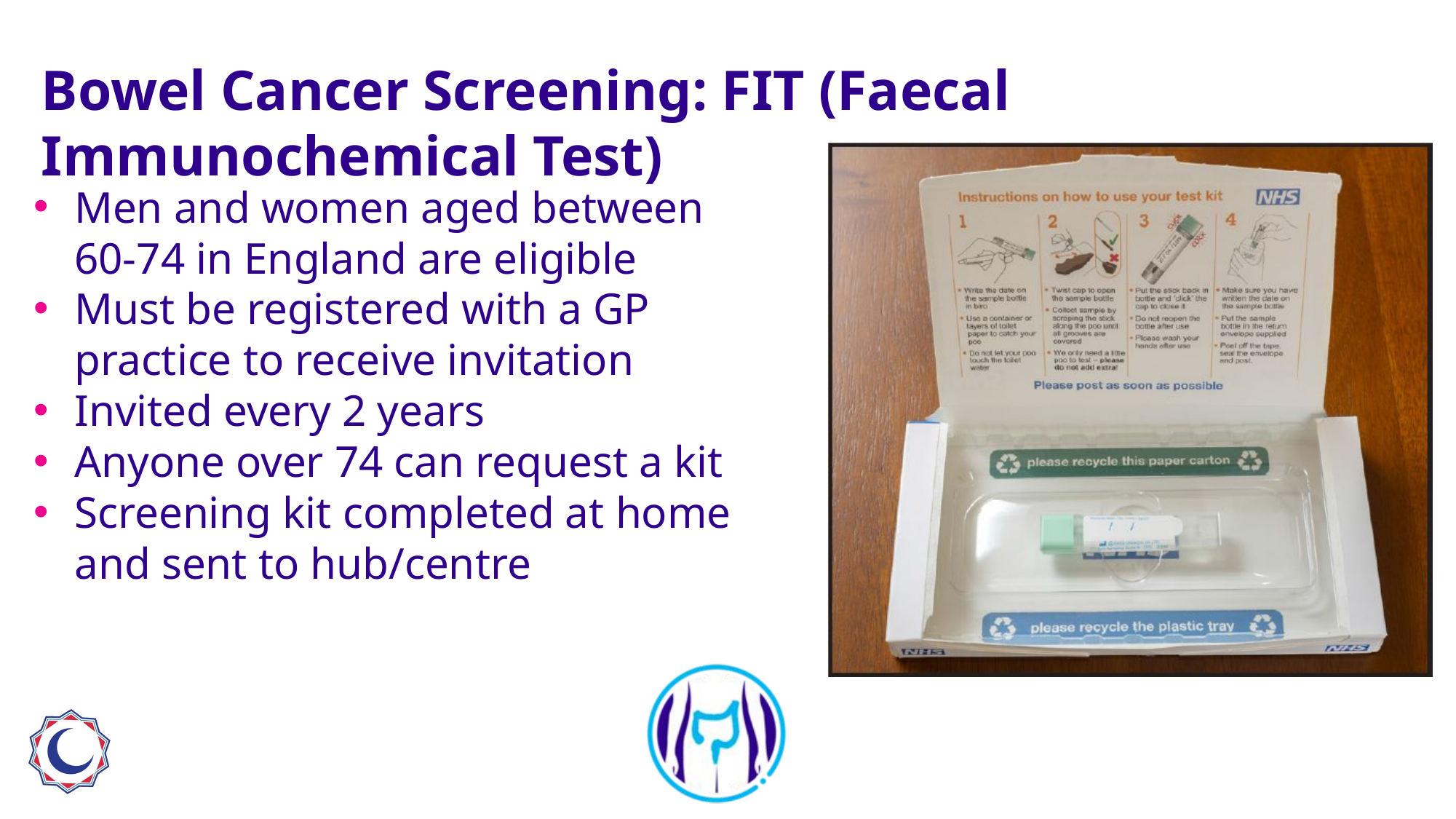

Bowel Cancer Screening: FIT (Faecal Immunochemical Test)
Men and women aged between 60-74 in England are eligible
Must be registered with a GP practice to receive invitation
Invited every 2 years
Anyone over 74 can request a kit
Screening kit completed at home and sent to hub/centre

## Slide 13
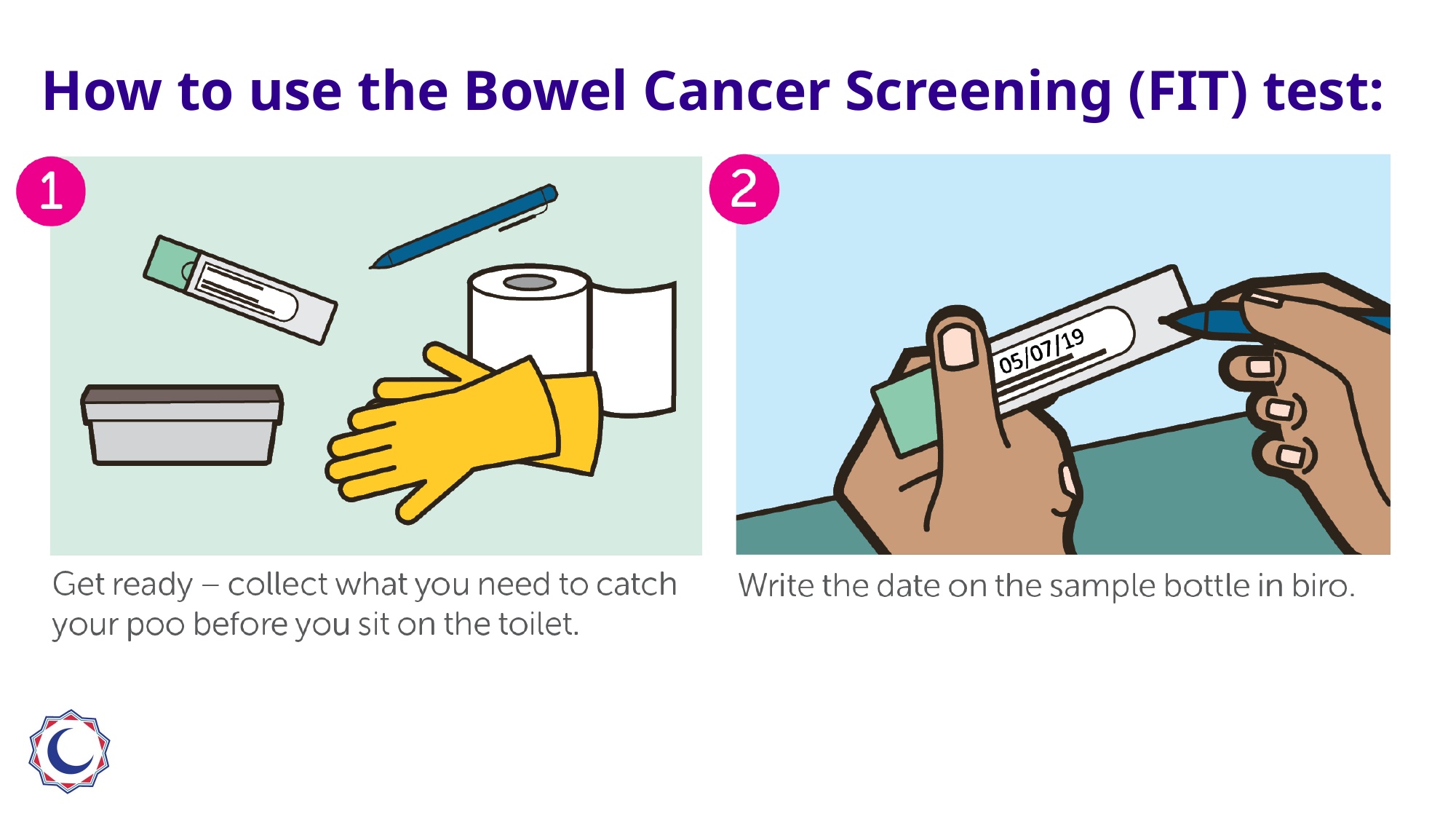

How to use the Bowel Cancer Screening (FIT) test:

## Slide 14
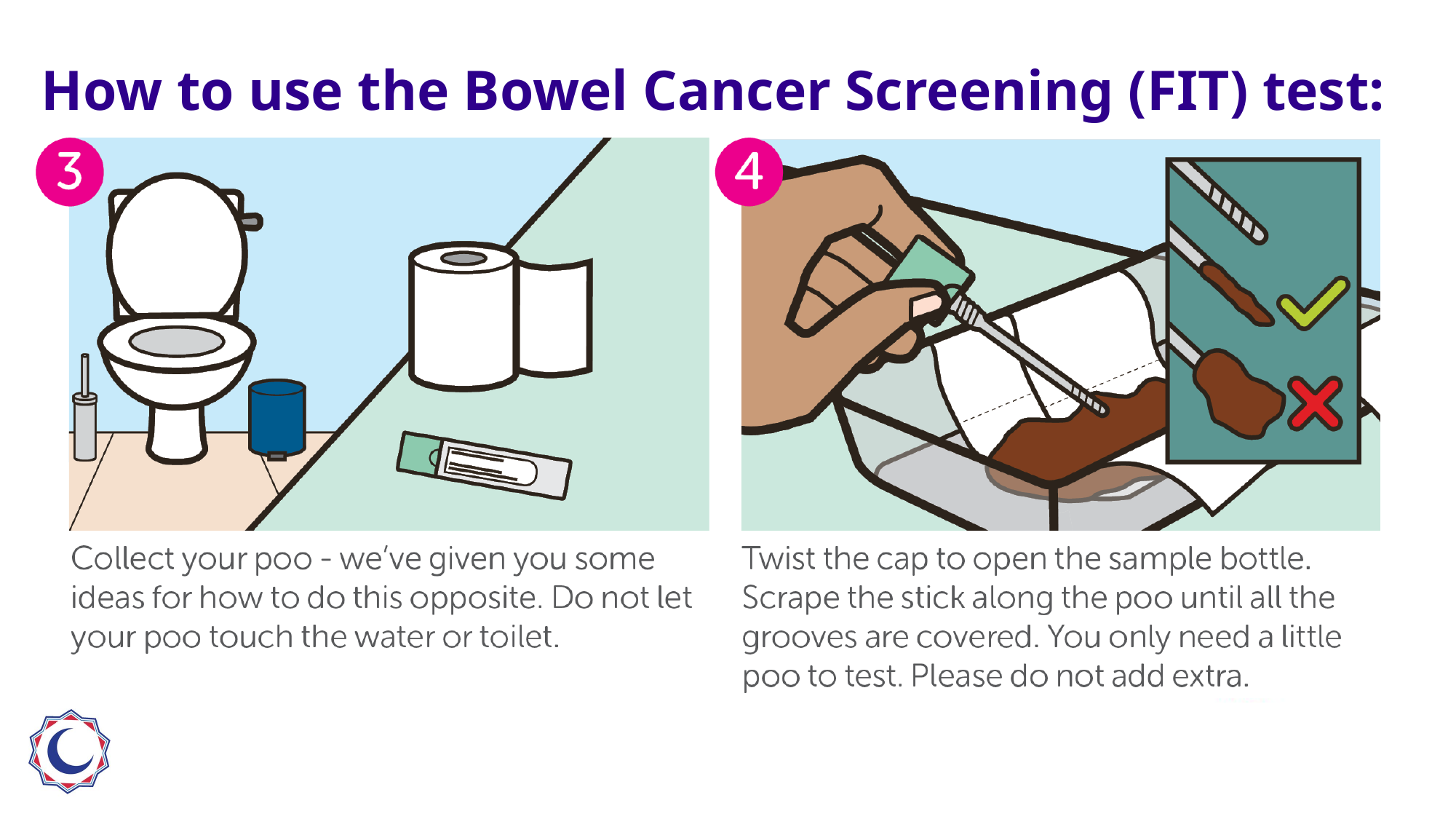

How to use the Bowel Cancer Screening (FIT) test:

## Slide 15
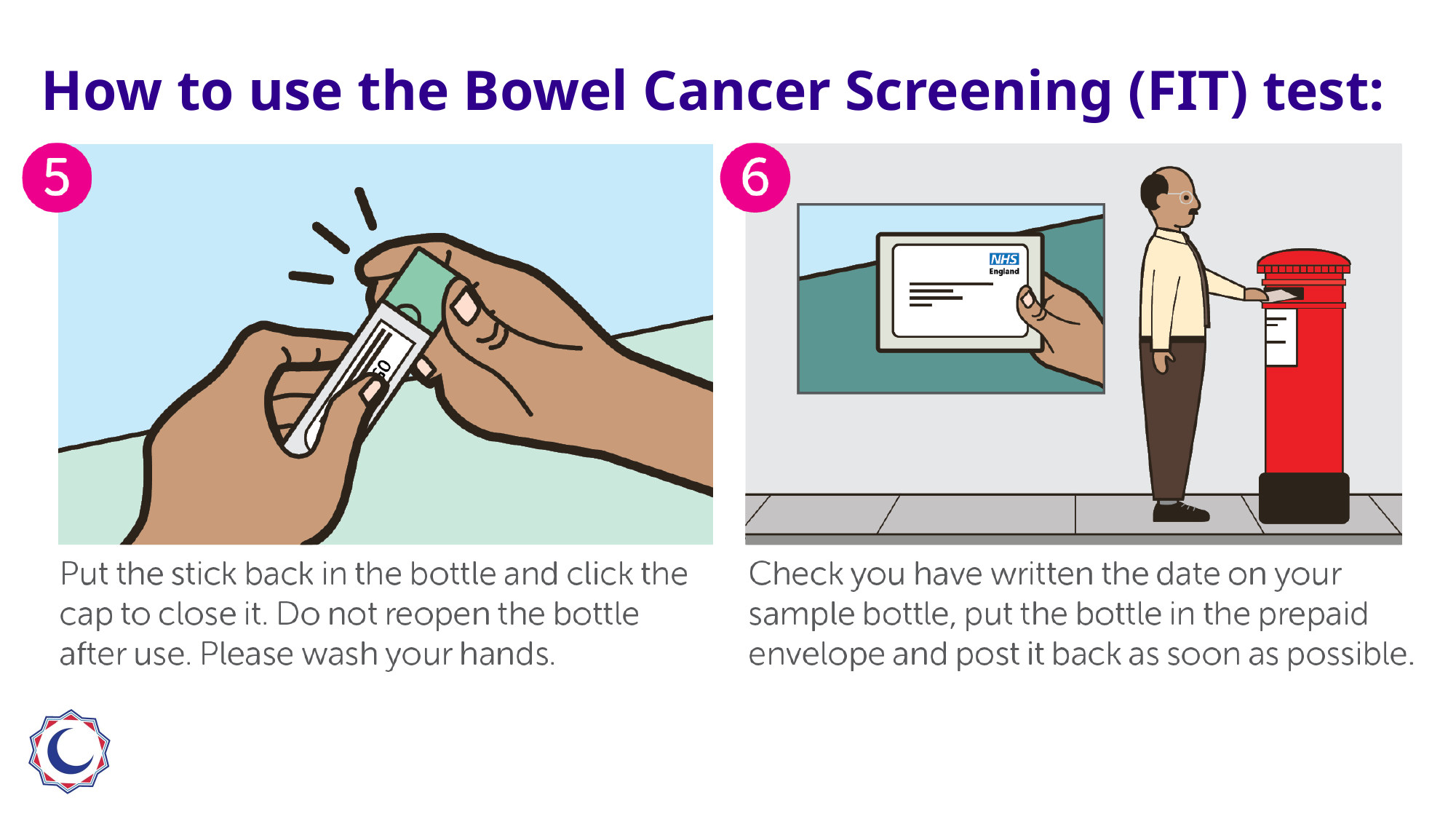

How to use the Bowel Cancer Screening (FIT) test:

## Slide 16
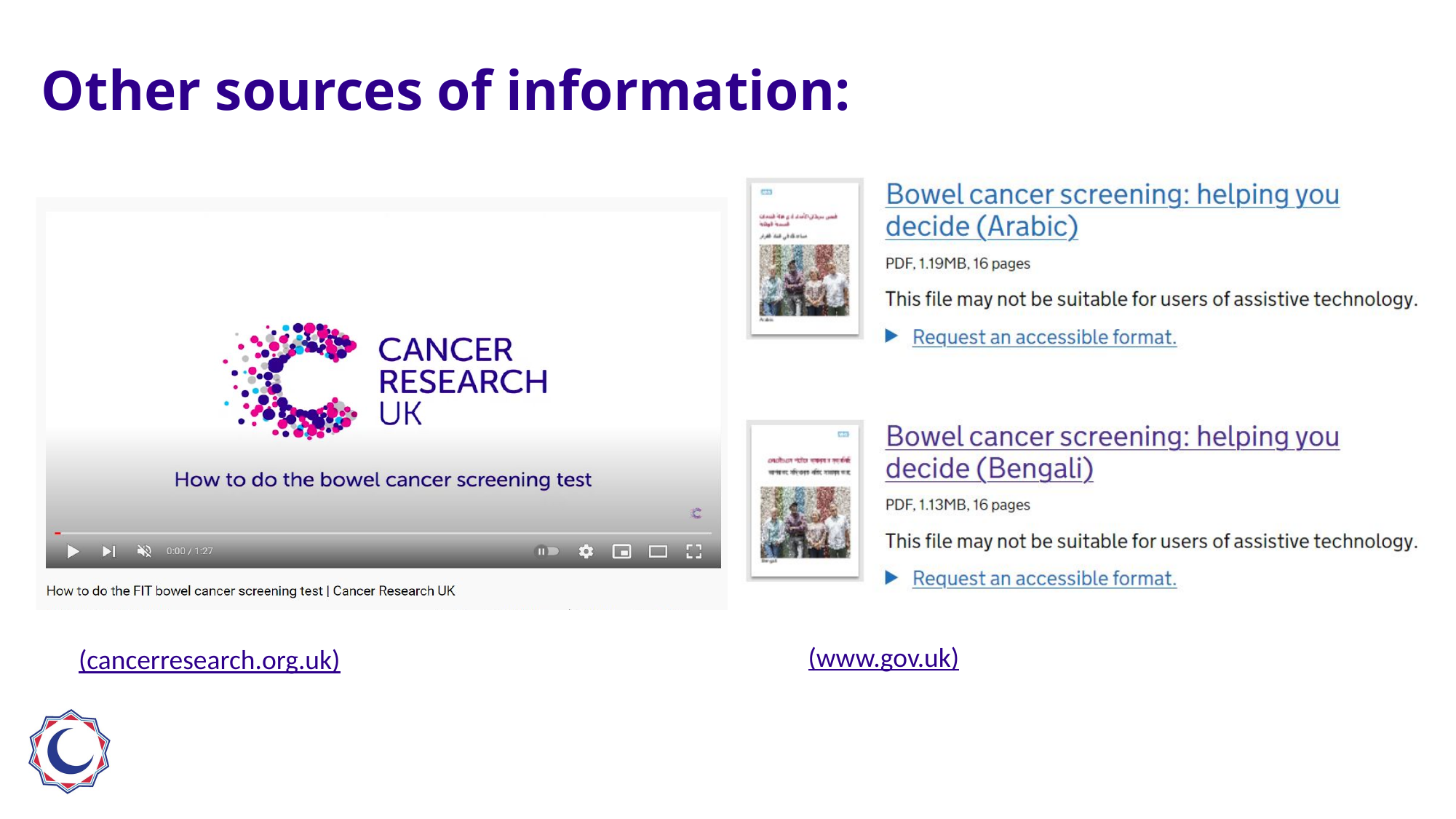

Other sources of information:
(www.gov.uk)
(cancerresearch.org.uk)

## Slide 17
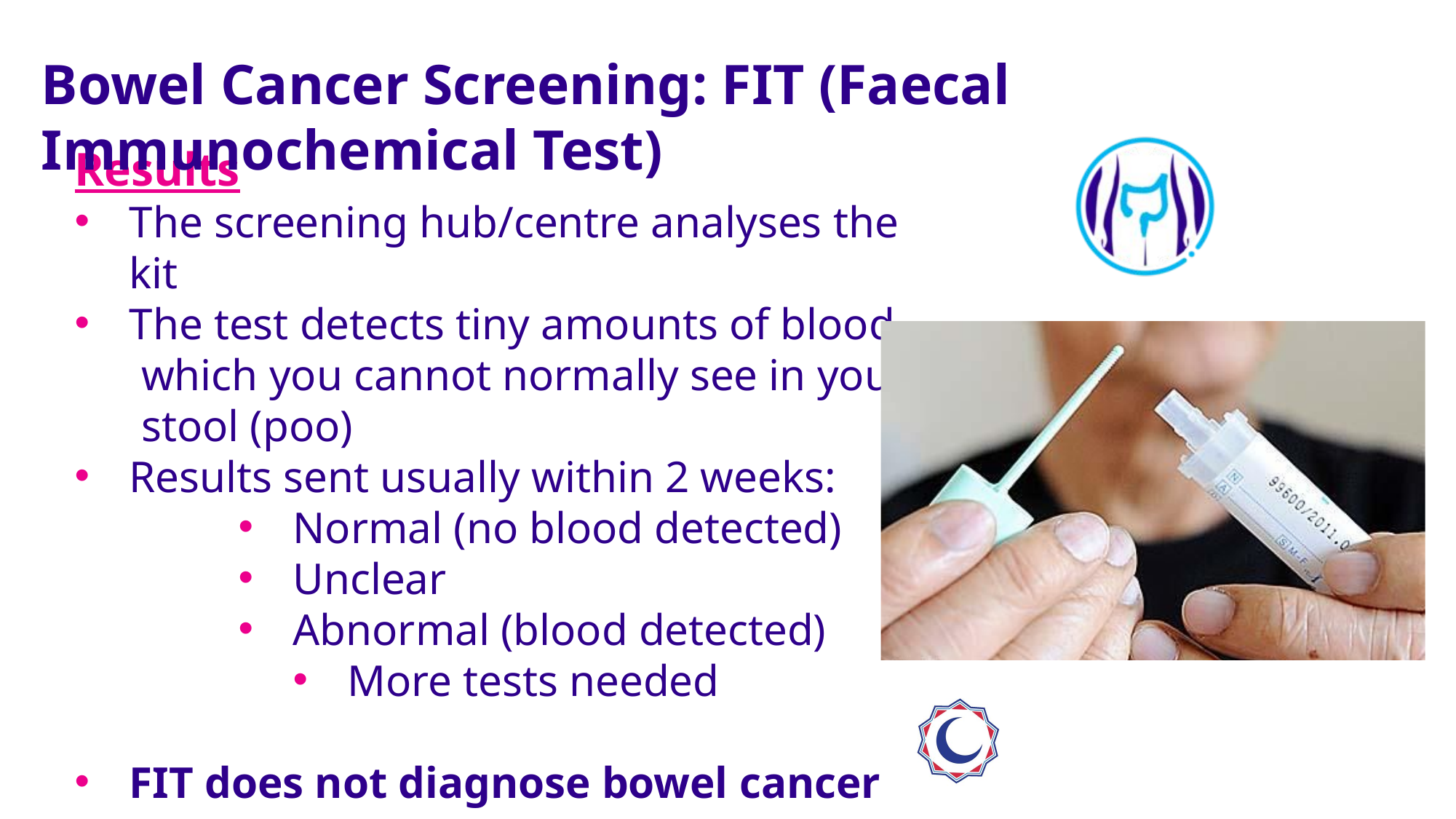

Bowel Cancer Screening: FIT (Faecal Immunochemical Test)
Results
The screening hub/centre analyses the kit
The test detects tiny amounts of blood
 which you cannot normally see in your
 stool (poo)
Results sent usually within 2 weeks:
Normal (no blood detected)
Unclear
Abnormal (blood detected)
More tests needed
FIT does not diagnose bowel cancer

## Slide 18
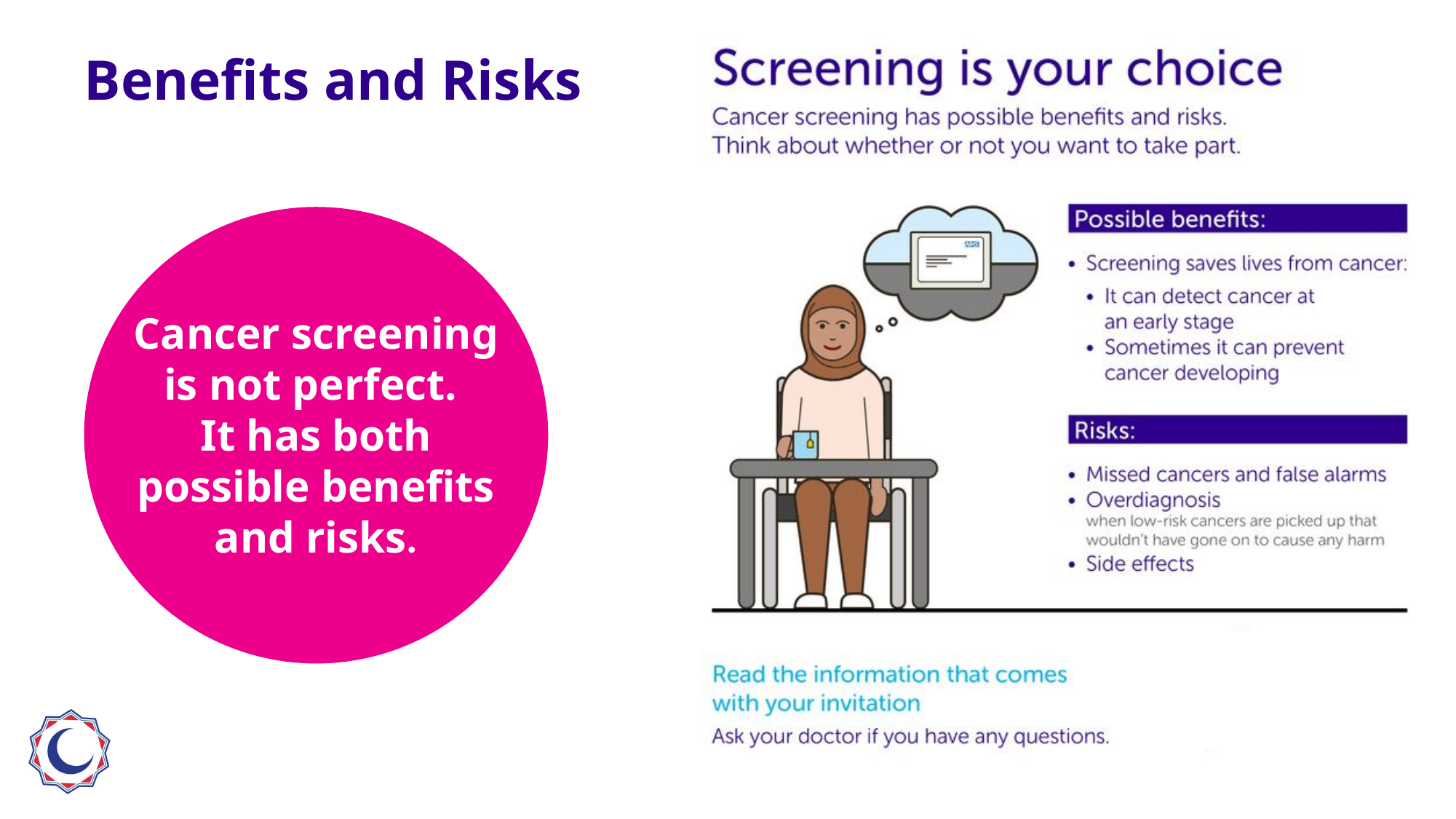

# Benefits and Risks
Cancer screening is not perfect.
It has both possible benefits and risks.

## Slide 19
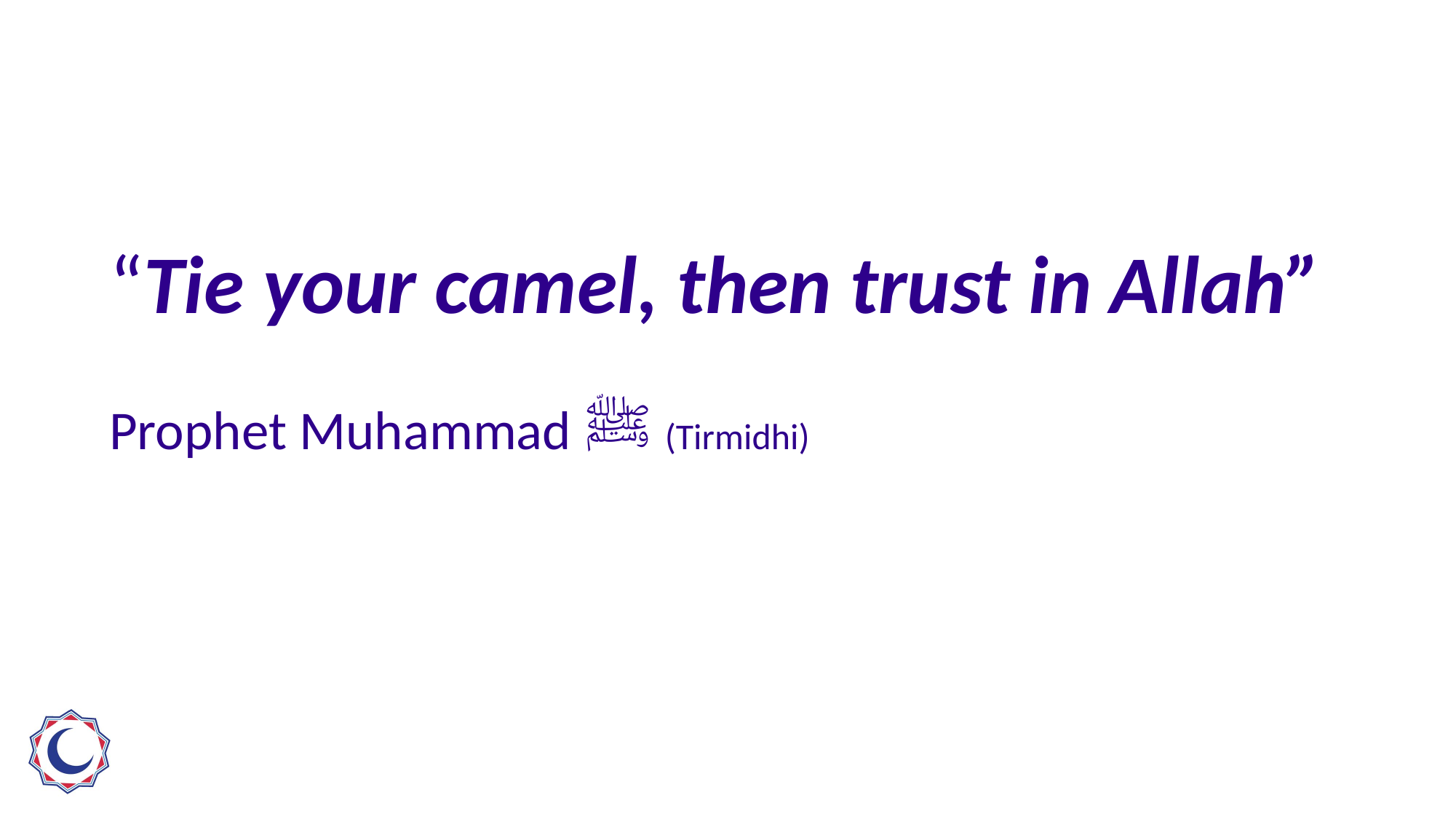

“Tie your camel, then trust in Allah”
Prophet Muhammad ﷺ (Tirmidhi)

## Slide 20
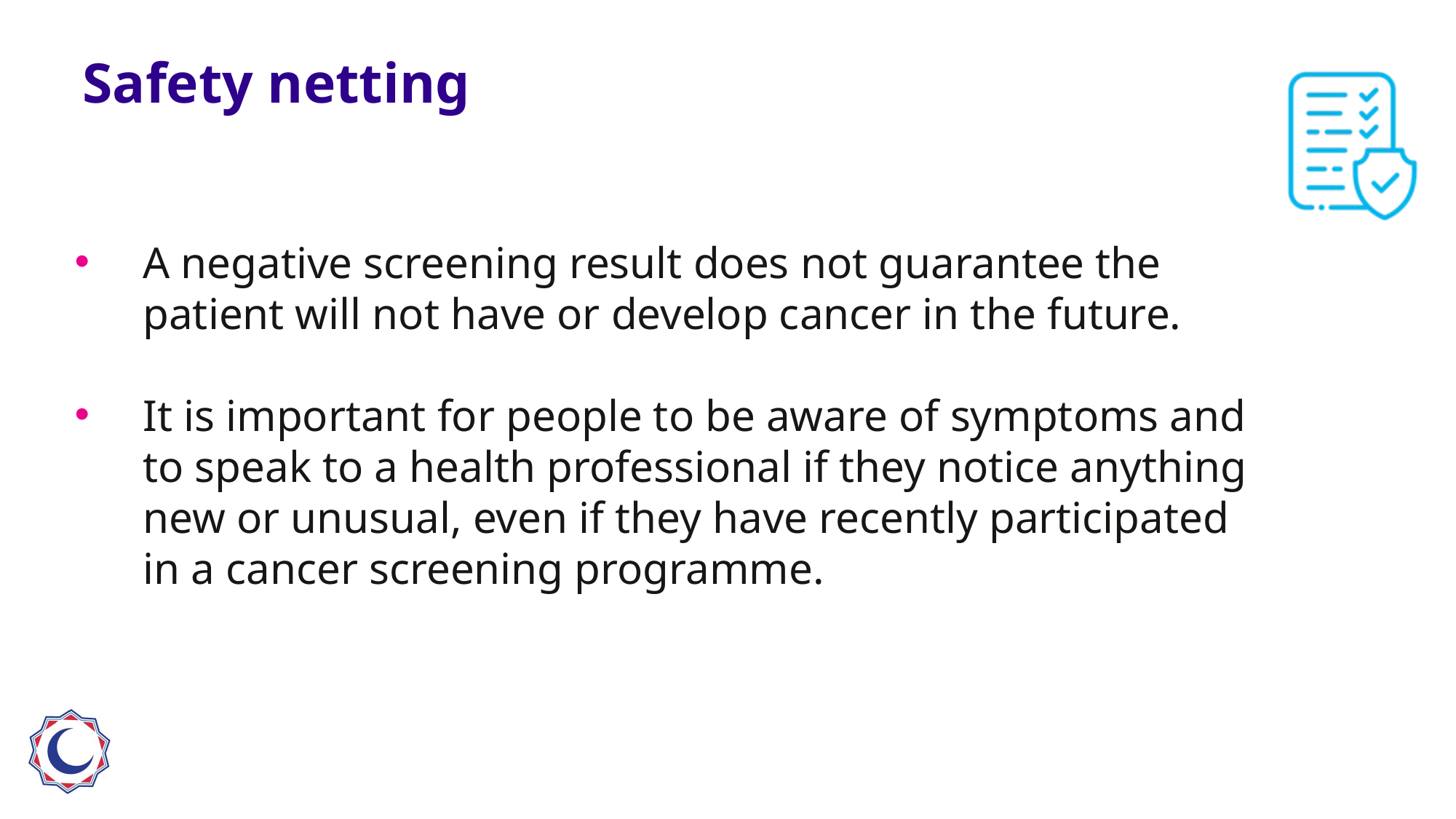

Safety netting
A negative screening result does not guarantee the patient will not have or develop cancer in the future.
It is important for people to be aware of symptoms and to speak to a health professional if they notice anything new or unusual, even if they have recently participated in a cancer screening programme.

## Slide 21
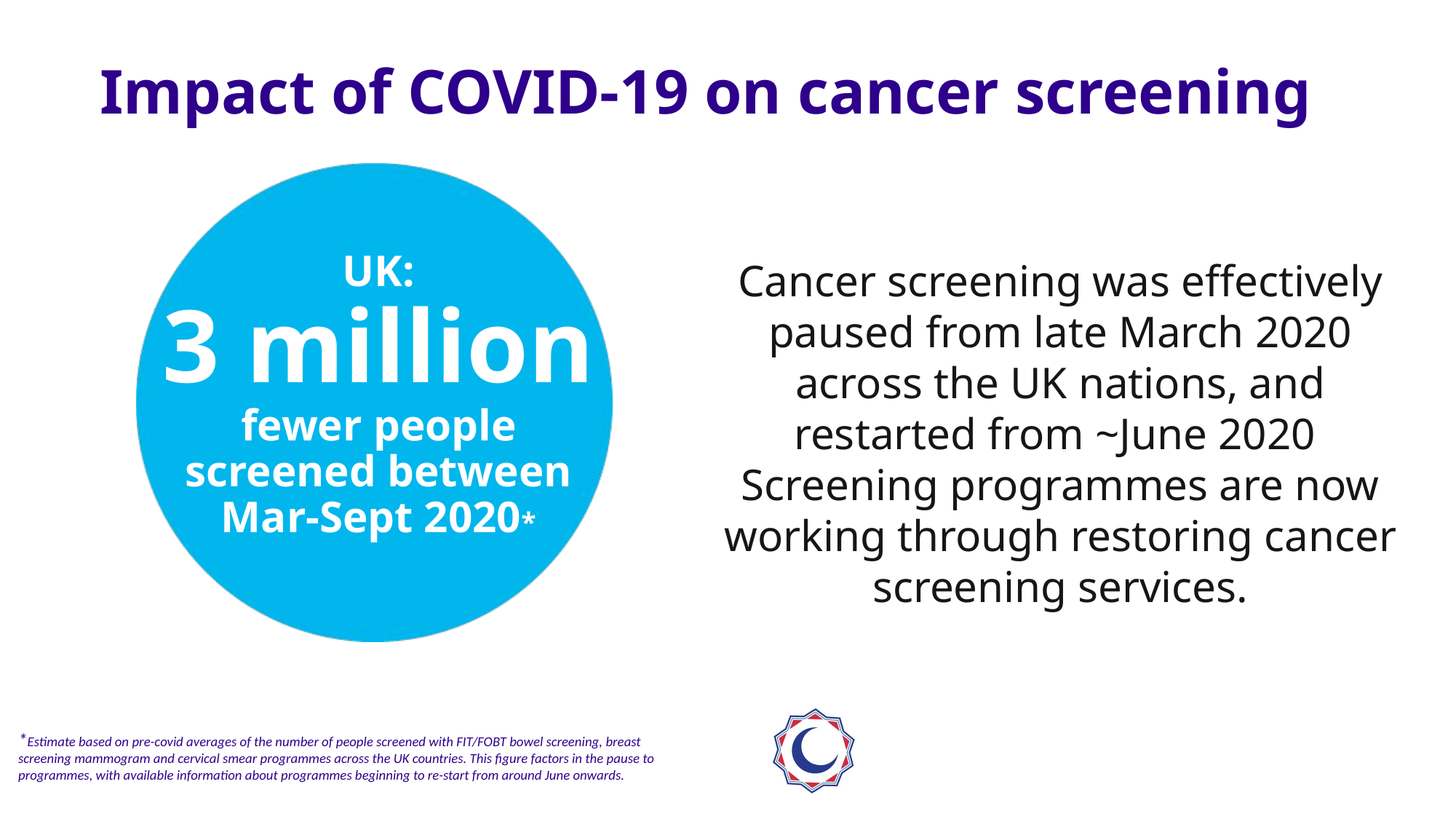

Impact of COVID-19 on cancer screening
UK:
3 million
fewer people screened between
Mar-Sept 2020*
Cancer screening was effectively paused from late March 2020 across the UK nations, and restarted from ~June 2020
Screening programmes are now working through restoring cancer screening services.
*Estimate based on pre-covid averages of the number of people screened with FIT/FOBT bowel screening, breast screening mammogram and cervical smear programmes across the UK countries. This figure factors in the pause to programmes, with available information about programmes beginning to re-start from around June onwards.

## Slide 22
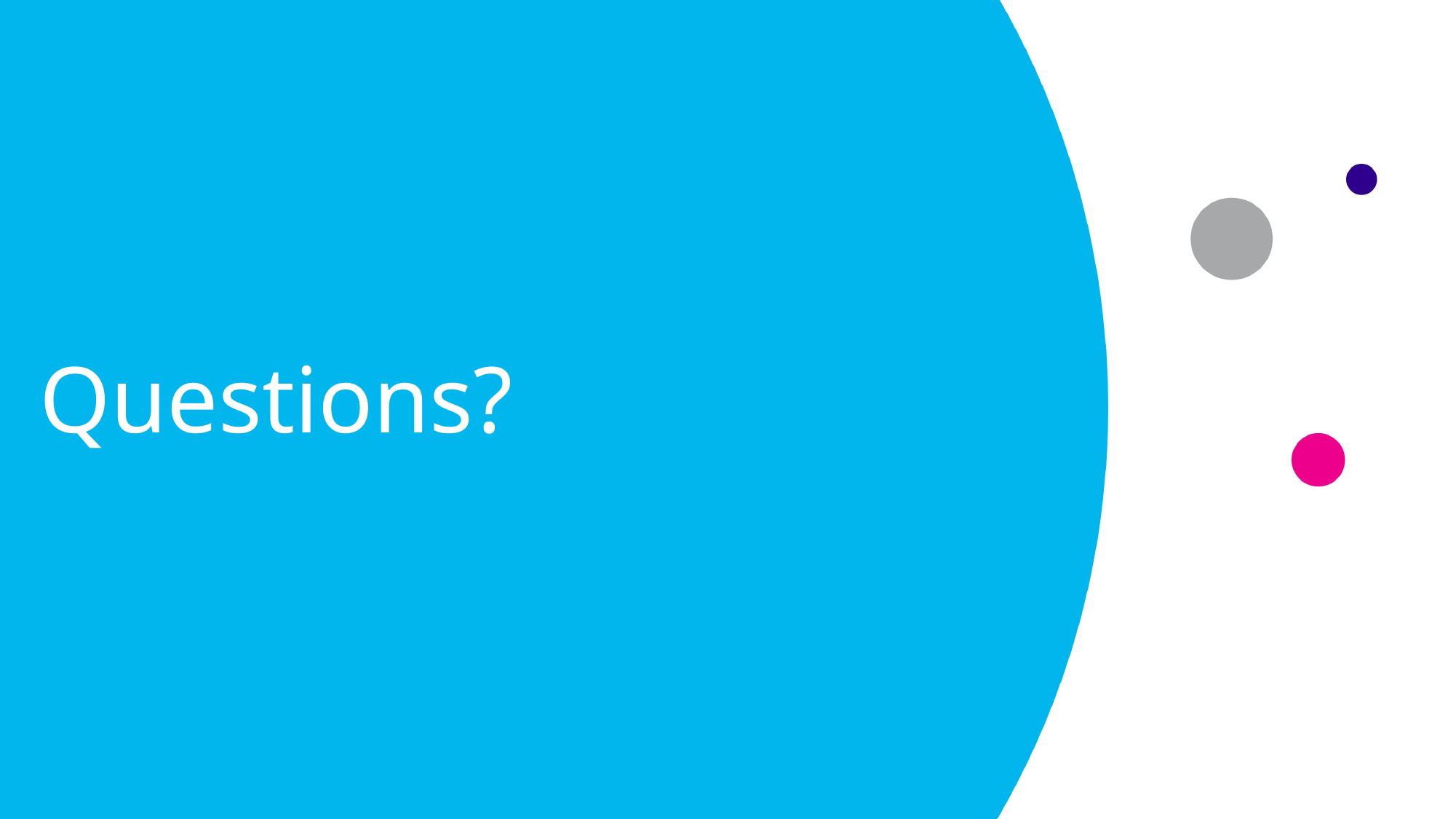

# Questions?
